# The role of critical immune genes in brain disorders: insights from neuroimaging immunogenetics

**DOI:** 10.1093/braincomms/fcac078

**Published:** 2022-03-31

**Authors:** Beilei Bian, Baptiste Couvy-Duchesne, Naomi R. Wray, Allan F. McRae

**Affiliations:** 1 Institute for Molecular Bioscience, The University of Queensland, Brisbane, QLD 4072, Australia; 2 Paris Brain Institute, CNRS, INRIA, Paris, France; 3 Queensland Brain Institute, The University of Queensland, Brisbane, QLD, Australia

**Keywords:** human leukocyte antigen, complement component 4, killer cell immunoglobulin-like receptor, neuroimaging, genetic association

## Abstract

Genetic variants in the human leukocyte antigen and killer cell immunoglobulin-like receptor regions have been associated with many brain-related diseases, but how they shape brain structure and function remains unclear. To identify the genetic variants in *HLA* and *KIR* genes associated with human brain phenotypes, we performed a genetic association study of ∼30 000 European unrelated individuals using brain MRI phenotypes generated by the UK Biobank (UKB). We identified 15 *HLA* alleles in *HLA* class I and class II genes significantly associated with at least one brain MRI-based phenotypes (*P* < 5 × 10^−8^). These associations converged on several main haplotypes within the *HLA*. In particular, the human leukocyte antigen alleles within an ancestral haplotype 8.1 were associated with multiple MRI measures, including grey matter volume, cortical thickness (TH) and diffusion MRI (dMRI) metrics. These alleles have been strongly associated with schizophrenia. Additionally, associations were identified between HLA-DRB1*04∼DQA1*03:01∼DQB1*03:02 and isotropic volume fraction of diffusion MRI in multiple white matter tracts. This haplotype has been reported to be associated with Parkinson’s disease. These findings suggest shared genetic associations between brain MRI biomarkers and brain-related diseases. Additionally, we identified 169 associations between the complement component 4 (*C4*) gene and imaging phenotypes. We found that *C4* gene copy number was associated with cortical TH and dMRI metrics. No *KIR* gene copy numbers were associated with image-derived phenotypes at genome-wide threshold. To address the multiple testing burden in the phenome-wide association study, we performed a multi-trait association analysis using trait-based association test that uses extended Simes procedure and identified MRI image-specific associations. This study contributes to insight into how critical immune genes affect brain-related traits as well as the development of neurological and neuropsychiatric disorders.

## Teaser

Bian *et al.* report the associations between genetic variants in the major histocompatibility complex region and brain MRI phenotypes. This study identified shared genetic associations between brain MRI biomarkers and brain-related diseases. These results contribute to the insights into how the immune genes affect brain structure and brain-related diseases.

## Graphical Abstract

**Graphical abstract:**
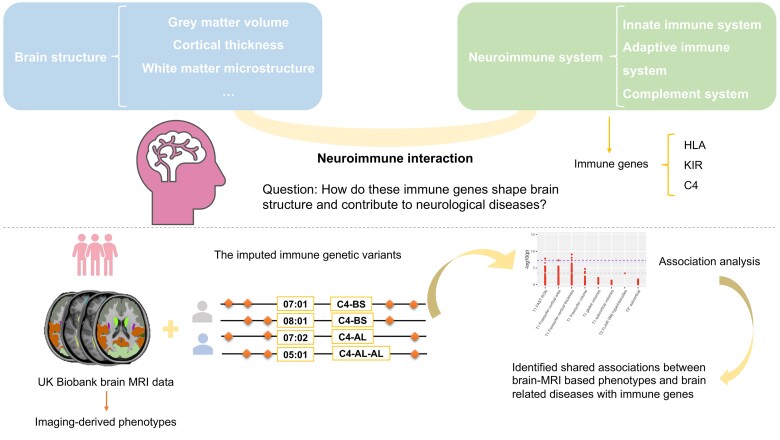

## Introduction

Brain MRI has been widely used for detecting the abnormalities of brain structure and function and contributes to the diagnostic process for different brain disorders.^1–4^ Previous studies have demonstrated that abnormalities in different brain regions are linked to different diseases. For instance, schizophrenia is associated with cortical grey matter deficits,^5^ and abnormalities of cerebral structure, such as the frontal pole and temporal pole, have been reported to be associated with bipolar disorder.^6^ With the advances in brain imaging, investigating white matter differences provides new opportunities to understand neurological diseases. Diffusion tensor imaging (DTI) studies have shown that the microstructure of white matter plays a role in Alzheimer’s disease, multiple sclerosis, amyotrophic lateral sclerosis and Parkinson’s disease.^7–10^ These findings support that MRI data can be used as intermediate phenotypes to discover new biomarkers for the clinical diseases and potentially elucidate disease pathways.

The large-scale datasets collected by the ENIGMA Consortium^11^ and the UK Biobank^12^ offer great opportunities to explore the genetic architecture of human brain structure and function. Genome-wide association studies (GWASs) conducted using UK Biobank data have identified a large number of genetic variants and genes associated with MRI measures.^13^ Further multi-phenotype association tests showed many of these identified genetic regions are also associated with mental illnesses and neurodegenerative diseases. Another notable study investigated the genetic contribution to variation between individuals in cerebral cortex traits, reporting a negative genetic correlation between surface area (SA) and cortical TH and that these traits are shaped by largely distinct loci.^14^

The major histocompatibility complex (MHC), also known as human leukocyte antigen (HLA) in human, and killer cell immunoglobulin-like receptor (KIR) have been associated with many neurological diseases.^15^ For instance, the *C4* gene in the HLA region has been associated with schizophrenia risk.^16^ Parkinson’s disease has been associated with *HLA* class I and class II loci and haplotypes.^17,18^*HLA-DR* and *HLA-DQ* genes were found to be associated with Alzheimer’s disease,^19,20^ while HLA-DRB1*15:01 was reported as the highest risk allele to be associated with MS.^21,22^ As far as we know, there is no genomic region as consistently associated with many brain-related diseases as the MHC region. However, association analyses of brain MRI phenotypes have provided only simple single nucleotide polymorphism (SNP)–based analysis, which missed the complexity of the MHC region. Here, we study imputed HLA haplotypes. Compared to the role of *HLA* in neurological diseases which has been studied over the past decades, the impact of variation in the KIR region has only been studied more recently. The *KIR* gene family is also a crucial component in regulation of the immune system, which is located on chromosome 19. This complex locus is characterized by high polymorphism in terms of copy number and haplotypes.^23^*KIR* genes encode a family of glycoproteins expressed on natural killer (NK) cells and a small number of subtypes of T cells. Most of the *KIR* genes encode inhibitory receptors which protect healthy cells from destruction by NK cells. Some of them are activating genes that can help remove the antigens like viruses and cancer. The combination of KIR and HLA class I ligands determines NK cell activation and inhibition, which may affect the outcome of infections.^24^ Few studies have investigated the association between KIR receptor/HLA ligand pairs and brain-related traits.

**Figure 1:**
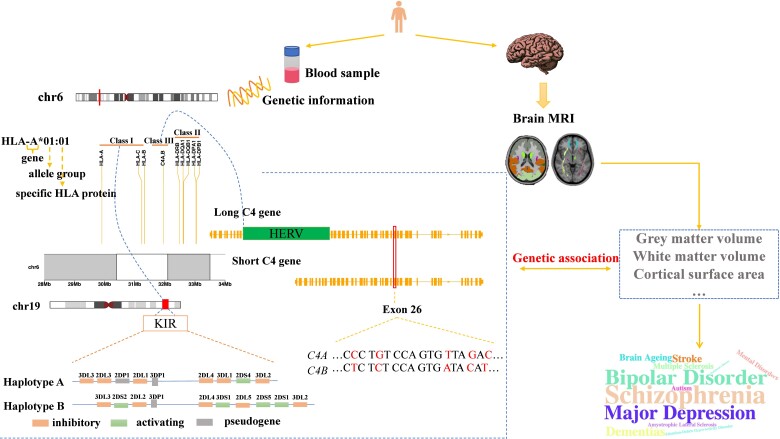
**The overview of the study. We identified 28 647 unrelated individuals of European descents from the UK Biobank.** These participants have both genotype data and brain MRI data. The brain imaging phenotypes were used as intermediate phenotypes. The genetic associations of the classical *HLA* genes, complement component 4 gene, *KIRs* as well as the interactions between *HLA* class I genes and *KIRs* were tested with 1422 IDPs. *HLA* gene family, located on chromosome 6p21.3, can be broadly divided into three classes: *HLA-A*, *HLA-B* and *HLA-C* are *HLA* class I genes. *HLA-DQA1*, *HLA-DQB1*, *HLA-DRB*, *HLA-DPA1* and *HLA-DPB1* are HLA class II genes. *C4* gene is an *HLA* class III gene, which has two isotypes, *C4A* and *C4B*. The long and short form of C4 gene can be distinguished by the presence or absence of the HERV sequence between exon 9 and exon 10. The difference between *C4A* and *C4B* lies in four amino acids encoded by exon 26. The *KIR* locus is located on the chromosome 19q13.4 and is characterized by a high degree of polymorphism in terms of copy number and haplotypes. The haplotypes can be broadly classified into haplotype A and haplotype B. Gene content in each haplotype is shown. The HLA class I molecules are the ligands for the KIR receptors.

The goal of this study is to investigate the relationship between the *HLA* and *KIR* loci and brain MRI-based phenotypes (Fig. 1). We conducted an association study using ∼30 000 unrelated individuals of European ancestry for who we had genotyping and brain MRI data in the UK Biobank. We tested the associations between 1422 image-derived phenotypes (IDPs) (Supplementary Table 1) and the imputed *HLA*, *KIR*, *C4* genes as well as KIR/HLA ligand pairs and the IDPs. We identified a large number of associations between immune genes and IDPs in the European population. Our study sheds light on how the immune genes may shape human brain development and contribute to neurological diseases.

## Materials and methods

### Brain imaging phenotypes data

We used IDPs released in January 2020 by UK Biobank. The UK Biobank acquired six MRI modalities (T1, fluid-attenuated inversion recovery (T2_FLAIR), susceptibility weighted imaging (SWI), dMRI, resting-state functional (rfMRI) and task-based functional (tfMRI)), consisting of structural, diffusion and functional imaging. The IDPs were generated from the raw images by using an imaging-processing pipeline developed by UK Biobank.^25^ We did not consider the rfMRI edges due to the low SNP-based heritability^13^ (h^2^ = 0.054, averaged over 1485 edges), which likely reflects a low test-retest reliability. In addition, we also included IDPs (volumes of subcortical structures, SA and cortical TH) estimated by FreeSurfer using Desikan–Killiany–Tourville atlas and Destrieux (a2009s) atlas, respectively. We organized these IDPs into nine groups (Supplementary Table 1) as suggested in the published GWAS paper.^13^ The basic quality control was performed by the UK Biobank in order to exclude the problematic MRI images.^25^ The description of imaging-processing protocol was summarized in the UK Biobank brain imaging documentation (https://biobank.ctsu.ox.ac.uk/crystal/crystal/docs/brain_mri.pdf).

Approximately 40 000 participants in UK Biobank have brain imaging phenotypes. Some of the IDPs have extreme distributions that may violate linear regression assumptions (the normality of the residuals). We calculated the skewness for each IDP (Supplementary Table 2). The skewness of each IDP was quite similar to that calculated in the previous published GWAS paper.^13^ To make sure the distribution of each IDP meet the linear regression assumptions, we used z-score calculated by rank-based inverse normal transformation (RINT) instead of raw IDP for each association analysis. To assess the robustness of the observed associations, we compared the HLA associations results between raw and transformed data using four IDPs with distinct distributions (Supplementary Fig. 1 and Supplementary Table 3). We observed similar results so we used RINT to carry out the association study. In our association analysis for each IDP, we ignored the individuals who had missing values.

### Genetic data

We used the raw genotype data released in July 2017 by UK Biobank. Genotyping was performed using Affymetrix UK BiLEVE Axiom array and the Affymetrix UK Biobank Axiom array. There are 456 426 individuals of European ancestry in UK Biobank V2 genotype data. We selected unrelated individuals (gcta^26^ –rel-cut-off 0.05) who have both genotype data and imaging-derived phenotypes. We removed the withdrawn individuals in UKB cohort from our study. This left a subset of 28 647 individuals identified for inclusion in the association study. We then extracted the specific regions covering *MHC* and *KIR* loci, respectively. These genotype data were used for *C4* and *KIR* genes imputations.

### Imputation

#### Imputed HLA alleles

HLA alleles were imputed by UK Biobank using HLA*IMP:02.^27^ The HLA genes were imputed to a multi-population reference panel. The UK Biobank imputations include all 11 *HLA* loci with all possible four-digit alleles. For each locus, the two possible alleles were assigned according to the posterior probabilities. We used the allelic dosage data provided by UK Biobank for the association analysis. *HLA* alleles with MAF < 0.01 were removed from downstream analysis.

#### C4 imputation

We used Beagle 5.1 to impute *C4* alleles against the reference panel^28^ publicly available in dbGaP under accession number phs000672.v1.p1 (see Supplementary Note). We first extracted those SNPs in MHC region (24Mb-34Mb on chromosome 6) using PLINK1.9^29^ and converted the plink format file into variant call format (VCF) file. We kept SNPs with genotype missingness < 0.05, HWE *P*-value < 10^−6^ and minor allele count > 5. Then, conform-gt tool was used to modify the VCF file to make sure the SNPs matched with the reference panel. There were 2991 SNPs overlapped with the reference panel and were used for *C4* imputation. We extracted the DS field in the resultant VCF file and used it as allelic dosage data. All possible *C4* structural types were imputed but only the common alleles were used in the association analyses. The resultant *C4* alleles were encoded using < H_n_a_b_h_s > where n is the total number of *C4* genes, a is the number of *C4A* genes, b is the number of *C4B* genes and h is the number of HERV copies, respectively. The ‘s’ here is used to distinguish the haplotypes which have same combination of < a, b, h > but different flanking SNP haplotypes. We then converted the encoded results to possible C4 structural alleles. For example, <H_1_0_1_0 > means this allele carries 1 *C4* gene and 1 *C4B* gene. The corresponding structural allele is C4-BS. But < H_2_1_1_1 > could be AL-BS, AS-BL, BL-AS or BS-AL. The haplotypes do not attempt to distinguish the order of A, B, L and S. We chose the same names as previous publication^16^ for such haplotypes. The frequency and haplotype name of each *C4* allele are listed in Supplementary Table 4. The C4A expression level was predicted as C4Aexpression = (0.47*AL) + (0.47*AS) + (0.2*BL).^16^

#### KIR imputation

We imputed KIR genes using KIR*IMP.^23^ KIR*IMP provides a UK reference panel with 301 SNPs and 479 haplotypes. We extracted chr19:53Mb-58Mb using PLINK 1.9.^29^ 221 SNPs overlapped with the reference panel on the UKB array. To gain more accuracy for *KIR* imputation, we uploaded the data to Michigan Imputation Server^30^ and imputed this region against the HRC panel.^31^ There were 267 SNPs overlapped with the KIR reference panel according to SNP positions. We compared the allele frequencies between our data and the reference data and manually removed those ambiguous SNPs. Finally, we identified 258 SNPs that have similar allele frequencies with the reference data, which can be used for imputation. As a sanity check, we compared gene and haplotype frequencies with other studies. Similar frequencies of *KIR* variants were observed across multiple studies (Supplementary Tables 5 and 6). The imputed *KIR* variants, including 17 *KIR* loci, fine-scaled haplotypes as well as general haplotypes were used for downstream association analysis.

### Confounding factors

We followed the published GWAS paper which suggested three categories of confounding factors in association analysis:^13^ (i) imaging confound; (ii) genetic ancestry; and (iii) height, weight and BMI. For the imaging confounds, age, sex, age^2^, age*sex, age^2^*sex, site, date, date^2^ were included as the covariates. In addition, we also included the location of the head (*x*-position, *y*-position, *z*-position), head motion from both resting fMRI and task-based fMRI and volumetric scaling factor for head size in linear regression. To account for genetic ancestry confounds, we included 40 genetic principal components computed by UKB as covariates in our analyses. Some body measures could also be confounders for IDPs. Here, we included three traditional confounds height, weight and BMI as covariates. To assess the potential collider bias in our analysis, we also performed association tests without the body measures (height, weight and BMI) as covariates with a selected phenotype (Supplementary Notes, Supplementary Fig. 2).

### Statistical analyses

#### Associations of *HLA* alleles with IDPs

All the confounding factors and *HLA* genotype dosage data were regressed to each IDP. No missing values were found with age, sex, site, date, height and weight. Other missing covariate values of imaging confounds were substituted by the mean value of the covariate. Each *HLA* allele was tested using multiple linear regression.

(1) $$\begin{array}{c} IDP_{z-\mathrm{score}}=\beta_{0}+\beta_{1}HLA_{\mathrm{dosage}}+\beta X+\varepsilon \end{array}$$

where *IDP*_*z*−score_ is the z-score of IDP transformed using RINT, *β*_0_ is the intercept of the linear model, *β*_1_ is the effect size of the *HLA* allele, *HLA*_dosage_ is the dosage of each *HLA* allele, *β* is the coefficients matrix of the covariates, *X* is the confounding factor matrix and ɛ is the residual of the model.

#### Associations of C4 haplotypes with IDPs

We only tested the five most common C4 haplotypes out of the 29 imputed C4 structural haplotypes in our association study. Each haplotype name with the corresponding C4 imputation result is listed in Supplementary Table 4. We summed the dosage data according to haplotype name and used this result for association analysis.

(2) $$\begin{array}{c} IDP_{z-\mathrm{score}}=\beta_{0}+\beta_{1}C4_{\mathrm{haplotype}}+\beta X+\varepsilon \end{array}$$

where terms are as in Equation (1), except for *C*4_haplotype_ indicates the dosage of each C4 haplotype.

#### Associations of *C4* copy number with IDPs

We counted the copy number of *C4A*, *C4B*, *C4L*, *C4S* gene as well as the total copy number of *C4* gene according to the *C4* imputation result. *C4A* expression level was predicted as described in previous study.^16^ We then regressed each *C4* gene copy number and *C4A* expression level with other covariates to each *z*-score of IDP and conducted association analysis by using linear regression.

(3) $$\begin{array}{rl} IDP_{z-\mathrm{score}} & =\beta_{0}+\beta_{1}(\#C4\;\mathrm{gene}\;or\;C4A\;\mathrm{expression})\\ & \;+\beta X+\varepsilon \end{array}$$

where terms are as in Equation (1), except for # *C*4 gene indicates the number of *C4* gene of different isotypes C4A and C4B and structural types C4L and C4S as well as total copy number of *C4*, *C*4*A* expression indicates the predicted *C4A* expression level.

#### Associations of *KIR* gene with IDPs

KIR*IMP imputed 19 genetic variants in total, consisting of general haplotypes, fine-scaled haplotypes as well as 17 *KIR* genes copy number. We converted each haplotype into a single variable and tested them independently. For each KIR haplotype,

(4) $$\begin{array}{c} IDP_{z-\mathrm{score}}=\beta_{0}+\beta_{1}KIR_{\mathrm{haplotype}}+\beta X+\varepsilon \end{array}$$

where terms are as in Equation (1), except for *KIR*_haplotype_ is a variable counted the number of the specific haplotype each individual carried.

For each *KIR* gene copy number,

(5) $$\begin{array}{c} IDP_{z-\mathrm{score}}=\beta_{0}+\beta_{1}KIR_{\mathrm{copy}-\mathrm{number}}+\beta X+\varepsilon \end{array}$$

where terms are as in Equation (1), except for *KIR*_copy-*number*;_ is the copy-number of *KIR* gene.

#### Associations of epistatic interaction between KIR and HLA ligands with IDPs

KIR genes and their HLA ligands were listed in Supplementary Table 7. These ligand-receptor pairs have been previously validated experimentally.^32^ For the subgroup of HLA, we summed the HLA dosage data over four-digit HLA alleles belonging to each subgroup. We tested epistatic associations by introducing the interaction term KIR*HLA into the linear regression. The epistatic association analysis was carried out following the models used in a recent KIR/HLA epistasis study in ankylosing spondylitis disease.^32^ Briefly, we used dominant (0 = gene absent, 1 = one or more gene copy) and recessive (0 = dosage less than two, 1 =homozygosity at locus) models for KIR genes, respectively. HLA alleles were treated as having dominant effect.

(6) $$\;\begin{array}{rl} IDP_{z-\mathrm{score}} & =\beta_{0}+\beta_{1}KIR_{\mathrm{dominant}/\mathrm{recessive}}+\beta_{2}HLA_{\mathrm{dominant}}\\ & \;+\beta_{3}KIR_{\mathrm{dominant}/\mathrm{recessive}}\;*\;HLA_{\mathrm{dominant}}+\beta X+\varepsilon \end{array}$$

where *KIR*_dominant/recessive_ is the KIR status under dominant or recessive model, *HLA*_dominant_ is the HLA status under the dominant model, $KIR_{\mathrm{dominant}/\mathrm{recessive}}*HLA_{\mathrm{dominant}}$ is the interaction term under KIR dominant or recessive model.

### Correction for multiple testing

For single variant association analysis, we used a conservative *P* value threshold of 2.5 × 10^−7^ (0.05/(137*1422)) which accounted for the number of both genetic variants and IDPs tested in this study. We also considered a relaxed threshold of 3.6×10^−4^ (0.05/137), which only accounted for the number of genetic variants tested for each IDP. For KIR/HLA epistatic associations, we adjusted 0.05 significant threshold with the number of KIR/HLA pairs and IDPs (*P* < 3.1 × 10^−7^ (0.05/(112*1422)). The conservative thresholds were very close to the genome-wide significant threshold, so we also report the associations at the threshold of 5 × 10^−8^. These thresholds are conservative considering the correlation across IDPs and the strong linkage disequilibrium (LD) between neighbouring loci in HLA and KIR region, but these associations results should be interpreted carefully.

### Multi-trait association analysis

The multi-trait association analysis was carried out using TATES.^33^ We did not choose popular methods such as MTAG^34^ and Genomic SEM^35^ for the multivariate analysis considering that they are built on LD score regression,^36^ which excluded the MHC region. TATES is more flexible and allows users to use *P*-values as input and detects both genetic variants that are common to multiple phenotypes and genetic variants that are specific to a single phenotype. Thus, it is more appropriate for this study. In multi-trait association analysis, the IDPs were grouped into 19 classes (Supplementary Table 8). We only considered the additive effect between HLA, C4 and KIR and each IDP group. In brief, we first calculated the phenotypic correlation matrix after correcting for all the covariates used in univariate association analysis. We then corrected for the correlation matrix and acquired TATES *P*-values for each IDP group.

### Data availability

This study makes use of individual-level genotype and brain MRI phenotype data from UK Biobank Resource (application number: 12505). UK Biobank: https://www.ukbiobank.ac.uk.

## Results

### Summary

Imputation of four-digit HLA alleles from SNPs was carried out using HLA*IMP:02^27^ by the UK Biobank team (https://biobank.ctsu.ox.ac.uk/crystal/crystal/docs/HLA_imputation.pdf). The *C4* haplotypes were imputed using a mixed ancestry reference panel,^28^ with the *C4* copy numbers calculated from the imputed haplotypes. The KIRs including 17 *KIR* genes, KIR general haplotypes (A/B) as well as fine-scale haplotypes were imputed using KIR*IMP.^23^ We performed the genetic association analysis between the common genetic variants and IDPs using an additive genetic model. We considered three *P*-value thresholds with Bonferroni correction (see Materials and methods). At the most stringent threshold, the genome-wide threshold (5 × 10^−8^), we identified 299 associations between single genetic variants and IDPs (Supplementary Tables 9–11). The detailed association results at the other two thresholds, which accounted for both the number of variants and IDPs tested and the number of variants tested only, are shown in Supplementary Tables 12–18. The number of the significant associations at different thresholds is summarized in Supplementary Table 19. We observed shared associations across IDPs. The QQ-plots of the HLA associations with all IDPs and each category of IDPs are displayed in Supplementary Fig. 3, showing some inflation of test statistics. To demonstrate the inflation was not due to other confounding factors, we performed standard GWAS on five selected IDPs (Supplementary Note). These GWAS QQ-plots (Supplementary Fig. 4) and LD score regression intercepts (Supplementary Table 20) for these five IDPs show no other bias exists. These results indicate that the observed inflation in test statistics in our analysis is due to the correlation across *HLA* alleles as well as IDPs.

### Significant *HLA* alleles associated with IDPs

**Figure 2:**
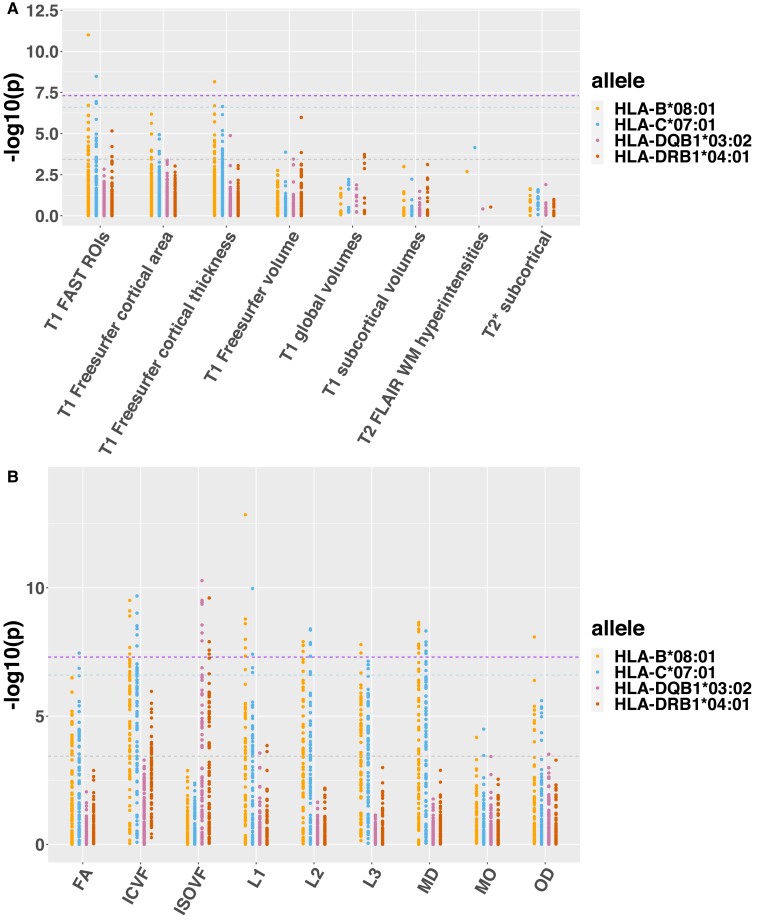
**PheWAS plots of selected HLA alleles across IDPs.** The IDPs were generally split into structural and dMRI groups. Structural IDPs were divided into eight subgroups, including T1 FAST region of interest (ROIs), T1 freesurfer cortical area, T1 freesurfer cortical TH, T1 freesurfer volume, T1 global volumes, T1 subcortical volumes, T2 FLAIR WM hyperintensities and T2* subcortical. dMRI IDPs were divided into nine subgroups, including FA, ICVF, ISOVF, L1, L2, L3, MD, MO and OD. Different subgroups of IDPs are given along the *x*-axis. The strength of association (−log_10_(*P* value)) for individual IDP within each subgroup is given on the *y*-axis. The purple dash line indicates the genome-wide significant −log_10_(*P* value) threshold of 7.3. The blue dash line indicates the conservative −log_10_(*P* value) threshold of 6.6 accounts for the number of loci (137) as well as total IDPs (1422) tested. The grey dash line indicates a nominal significance −log_10_(*P* value) threshold of 3.44 correcting only for the number of the tested loci (137). (**A**) The PheWAS plot of the association between structural IDPs and HLA-B*08:01, HLA-C*07:01, HLA-DQB1*03:02 and HLA-DRB1*04:01. (**B**) The PheWAS plot of the association between dMRI IDPs and HLA-B*08:01, HLA-C*07:01, HLA-DQB1*03:02 and HLA-DRB1*04:01.

We identified 15 *HLA* alleles associated with at least one IDP (*P* < 5×10^−8^), containing 130 associations. Using the genome-wide threshold, we did not find any significant associations with fMRI. This was consistent with the low SNP-based heritability of fMRI phenotypes^13^ which might be due to the low test-retest reliability of fMRI measurements.^37^ The PheWAS plot (Fig. 2 and Supplementary Figs. 5 and 6) shows the associations of 15 significant *HLA* alleles with structural and dMRI IDPs. The most significant associations of these 15 *HLA* alleles with associated IDPs are given in Table 1.

**Table 1:** Summary of the most significant HLA alleles with the associated IDPs

HLA allele name	Top association with structural MRI (*P* < 5 × 10^−8^)	Top association with dMRI (*P* < 5 × 10^−8^)	Direction of effect (structural MRI/dMRI)
HLA-A*01:01	None	Mean MD in posterior thalamic radiation on FA skeleton (left)	None/−
HLA-B*07:02	None	Mean L1 in cingulum hippocampus on FA skeleton (left)	None/−
HLA-B*08:01	Volume of grey matter in thalamus (left)	Mean L1 in superior longitudinal fasciculus on FA skeleton (left)	−/−
HLA-C*05:01	None	Mean ICVF in cerebral peduncle on FA skeleton (right)	None/−
HLA-C*07:01	Volume of grey matter in thalamus (left)	Mean L1 in superior longitudinal fasciculus on FA skeleton (left)	−/−
HLA-C*07:02	None	Mean OD in cerebral peduncle on FA skeleton (left)	None/+
HLA-DRB3*01:01	Mean TH of insula (left hemisphere)	None	+/None
HLA-DRB4*01:03	None	Weighted-mean ISOVF in tract superior longitudinal fasciculus (right)	None/+
HLA-DRB1*03:01	Mean TH of insula (left hemisphere)	Mean L2 in posterior thalamic radiation on FA skeleton (left)	+/−
HLA-DRB1*04:01	None	Weighted-mean ISOVF in tract inferior fronto-occipital fasciculus (left)	None/+
HLA-DRB1*04:04	None	Weighted-mean ISOVF in tract superior longitudinal fasciculus (right)	None/+
HLA-DQB1*02:01	Volume of grey matter in Thalamus (left)	Mean L2 in posterior thalamic radiation on FA skeleton (left)	−/−
HLA-DQB1*03:02	None	Weighted-mean ISOVF in tract superior longitudinal fasciculus (right)	None/+
HLA-DQA1*03:01	None	Weighted-mean ISOVF in tract superior longitudinal fasciculus (right)	None/+
HLA-DQA1*05:01	Area of S-pericallosal (right hemisphere)	None	−/none

L1,2 = in a diffusion tensor fit, the strength of diffusion along the principal axes of the ellipse (eigen values).

Overall, we observed some similarities in association patterns in terms of both P values and signs of effect across some HLA alleles. For example, we found HLA-A*01:01, HLA-C*07:01, HLA-B*08:01, HLA-DRB1*03:01/DRB3*01:01, HLA-DQB1*02:01 and HLA-DQA1*05:01 were associated with numerous structural and dMRI IDPs, including the regional grey matter volume, cortical TH, fractional anisotropy (FA), intra-cellular volume fraction (ICVF) and mean diffusivity (MD) (Fig. 2 and Supplementary Figs. 5 and 6). HLA-DRB1*04:01/04, HLA-DQA1*03:01, HLA-DQB1*03:02 and HLA-DRB4*01:03 were mainly associated with isotropic volume fraction (ISOVF) of dMRI which indicates free water volume fraction. In addition, HLA-B*07:02 and HLA-C*07:02 were associated with cortical SA in multiple regions at the nominal threshold, while strongly associated with orientation dispersion (OD) in cerebral peduncle and T2* in the hippocampus. HLA-C*05:01 was strongly associated with ICVF and OD in the cerebral peduncle (Supplementary Table 15).

**Figure 3:**
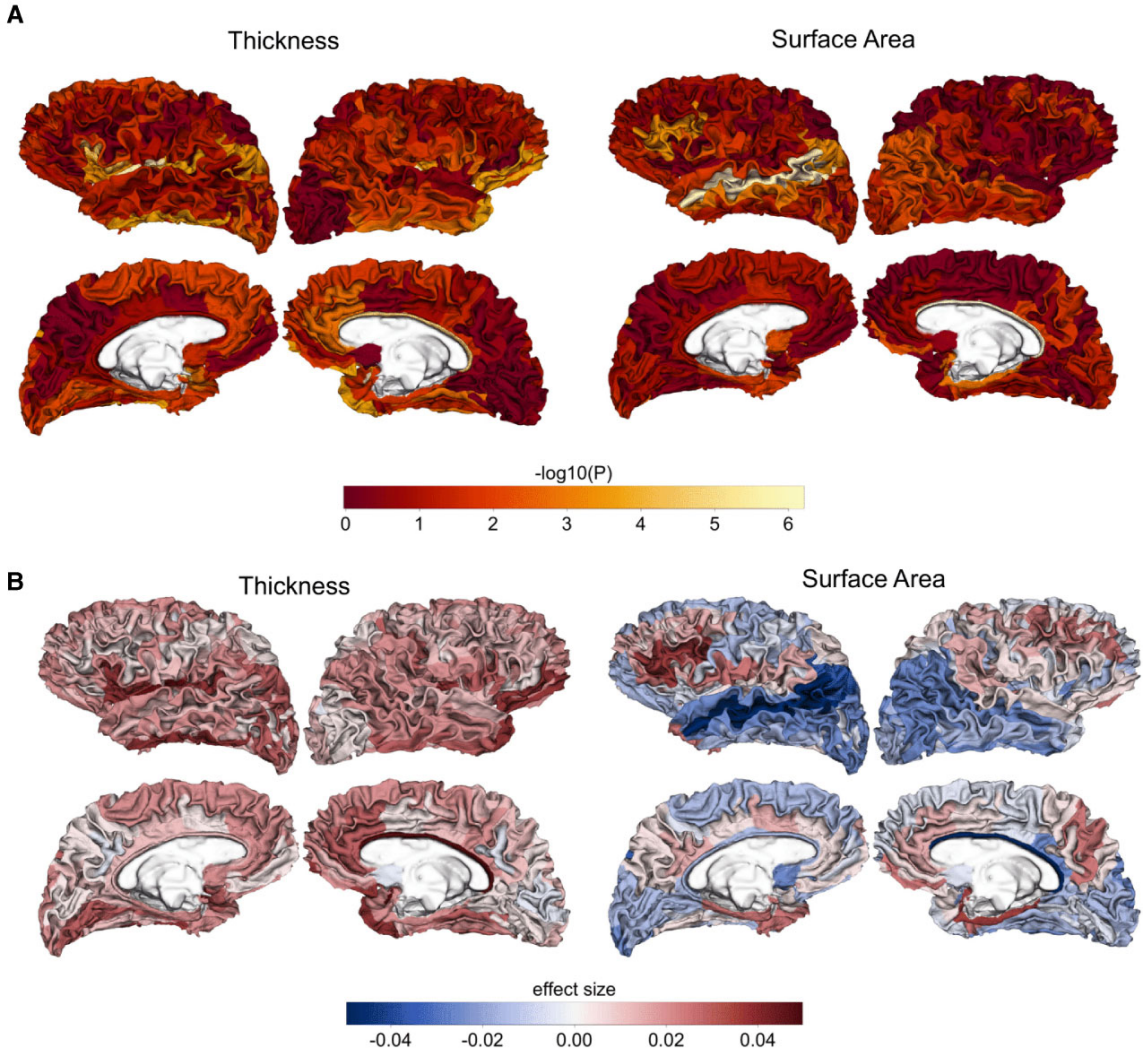
Spatial maps of the associations between HLA-B*08:01 and cortical SA and TH. (**A**) Spatial maps of the association between HLA-B*08:01 and SA and TH within whole brain. −log_10_(*P* values) are shown on freesurfer a2009s atlas with 74 regions included. (**B**) Spatial maps of the effect size of HLA-B*08:01 on TH and SA within whole brain.

We observed distinct *HLA* alleles affect cortical SA and TH. For instance, HLA-B*08:01 was associated with TH in several regions at the nominal threshold, while HLA-B*07:02 was associated with SA (Supplementary Table 15). The spatial distribution of −log_10_(P) values and effect sizes of HLA-B*08:01 and HLA-B*07:02 within the whole brain indicates the association strengths and effects on SA and TH (Fig. 3; Supplementary Fig. 7).

**Figure 4:**
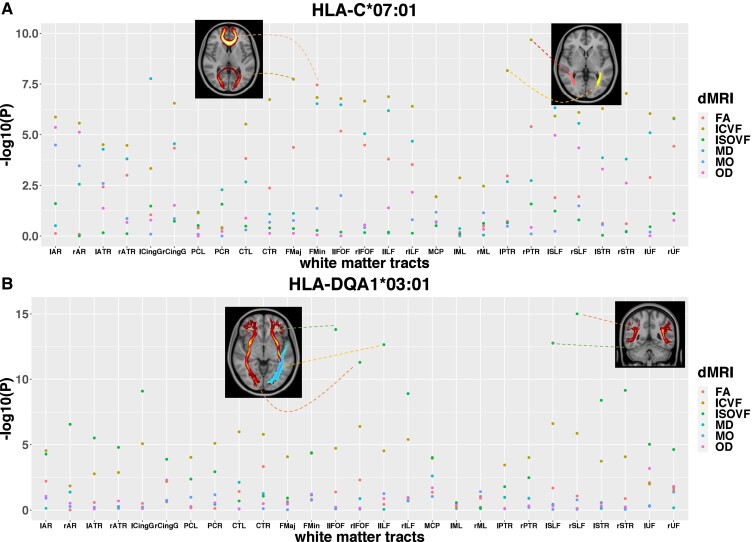
The associations of HLA-C*07:01 and HLA-DQA1*03:01 with dMRI metrics across white matter tracts in the brain. (**A**) The associations of HLA-C*07:01 with dMRI metrics across white matter tracts in the brain. (**B**) The associations of HLA-DQA1*03:01 with dMRI metrics across white matter tracts in the brain. The x-axis indicates the name of each white matter tract. The y-axis indicates the −log_10_(*P*-value). The selected significant associated white matter tracts are displayed on the standard MNI152 T1 image. AR, acoustic radiation; ATR, anterior thalamic radiation; CingG: cingulum gyrus; PCL/PCR, parahippocampal part of cingulum (left/right); CTL/CTR, corticospinal tract (left/right); FMaj, forceps major; FMin, forceps minor; IFOF, inferior fronto-occipital fasciculus; ILF, inferior longitudinal fasciculus; MCP, middle cerebellar peduncle; ML, medial lemniscus; MO, diffusion tensor mode; PTR, posterior thalamic radiation; SLF, superior longitudinal fasciculus; STR, superior thalamic radiation; UF, uncinate fasciculus; l, left; r, right.

**Figure 5:**
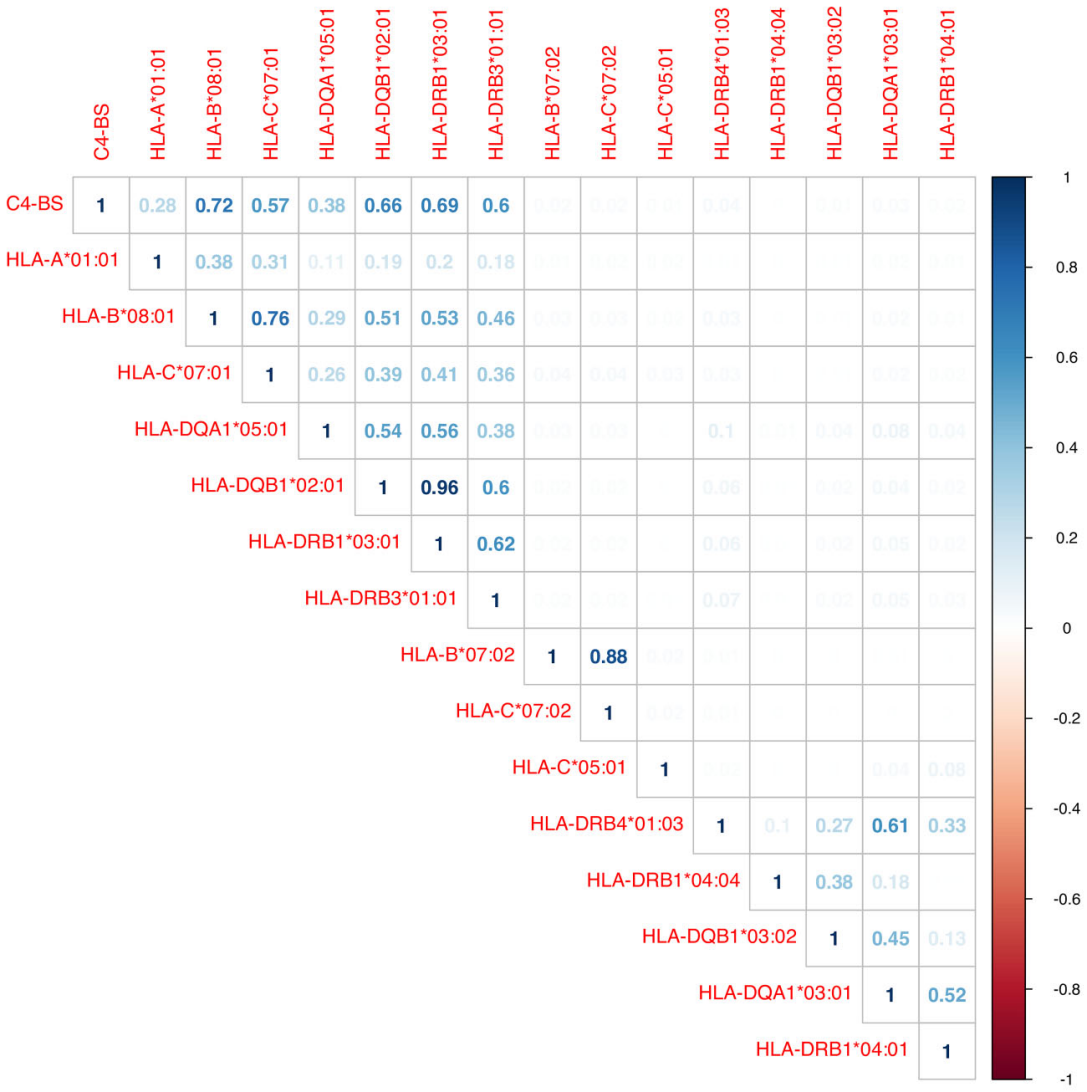
**Estimated LD across significant HLA alleles and C4-BS.** Only the upper triangular of the matrix is displayed. The rows and columns indicate the selected genetic variants. Dark blue indicates strong LD. The number in each cell indicates the LD measure between two genetic variants which is the squared correlation based on the genotype allele counts.

We investigated if the HLA haplotypes found here to be associated with IDPs have been reported to be associated with brain-related diseases. For example, HLA-B*08:01, HLA-DRB1*03:01 and HLA-DQB1*02 are associated with schizophrenia^16^ and depression.^38^ These alleles were found to be associated with a number of structural IDPs. Another example is the association between HLA-B*07:02 and Alzheimer’s disease.^39^ The significant association was found between HLA-B*07:02 and T2* in the hippocampus (essential for memory and a biomarker of Alzheimer's disease). Additionally, we observed distinct *HLA* alleles associated with different diffusion metrics (Fig. 2B and Supplementary Fig. 6). For example, HLA-C*07:01 (Fig. 4A) and other alleles in LD (Fig. 5) were mainly associated with FA of forceps minor, ICVF of posterior thalamic radiation, MD of cingulate gyrus tract (genetically correlated with major depression^40^). This long haplotype contains both MHC class I and II genes. MHCI molecules have been shown to be expressed on neurons. These proteins are crucial for synapses pruning,^41^ early brain development^42^ as well as ageing.^43^ In contrast, HLA-DRB1*04:01/04, HLA-DQA1*03:01, HLA-DQB1*03:02 and HLA-DRB4*01:03 were only associated with ISOVF across multiple tracts, including superior longitudinal fasciculus, inferior fronto-occipital fasciculus and inferior longitudinal fasciculus (Fig. 4B). Interestingly, these alleles have been repeatedly found to be associated with Parkinson’s disease.^17,18,44^ Taken together, these shared associations suggest that MHCI and MHCII molecules might be involved in multiple biological processes in the brain.

To test whether other SNPs in the MHC region were associated with IDPs, we selected five IDPs that were found to be significantly associated with distinct HLA alleles (Supplementary Table 21). We first performed the genetic association study with the SNPs in the MHC region (chr6: 29Mb-34Mb, covering *HLA-A*, *HLA-B*, *HLA-C*, *HLA-DQ*, *HLA-DP*, *HLA-DR* genes), identifying significant associations with SNPs located near or at the classical HLA genes. To further examine additional independent associations, we tested all the SNPs in the MHC region conditioning on all common classical four-digit *HLA* alleles of the associating *HLA* genes. For all five IDPs, we did not find any significant SNP after fitting the *HLA* allele (*P* < 5 × 10^−8^) (Supplementary Fig. 8), indicating the SNPs based association signals are not independent of the *HLA* alleles.

### Similarity across *HLA* alleles

Due to the complex linkage disequilibrium (LD) in MHC region, we observed several sets of *HLA* alleles that have similar association patterns across IDPs. To further investigate the similarity between those significant alleles, we calculated the squared correlation coefficient using the genotype count number of the *HLA* alleles (Fig. 5). From the correlation, we found HLA-A*01:01, HLA-B*08:01, HLA-C*07:01, HLA-DQA1*05:01, HLA-DRB1*03:01/DRB3*01:01 and HLA-DQB1*02:01 exhibit strong LD. This observation was quite similar with the association patterns (Fig. 2 and Supplementary Figs. 5 and 6). According to previous MHC haplotype studies,^45,46^HLA-A*01:01∼HLA-C*07:01∼HLA-B*08:01∼HLA-DQA1*05:01∼HLA-DRB1*03:01∼HLA-DQB1*02:01 is a common ancestral haplotype (also known as AH8.1, frequency = 0.09 in our study) in the European population. Similarly, HLA-C*07:02∼HLA-B*07:02 is a common HLA-C-B haplotype (frequency = 0.15 in our study). HLA-DQA1*03:01∼HLA-DQB1*03:02∼HLA-DRB1*04:01/04∼DRB4*01:03 is a class II haplotype.

### C4-BS was associated with numerous IDPs

**Figure 6:**
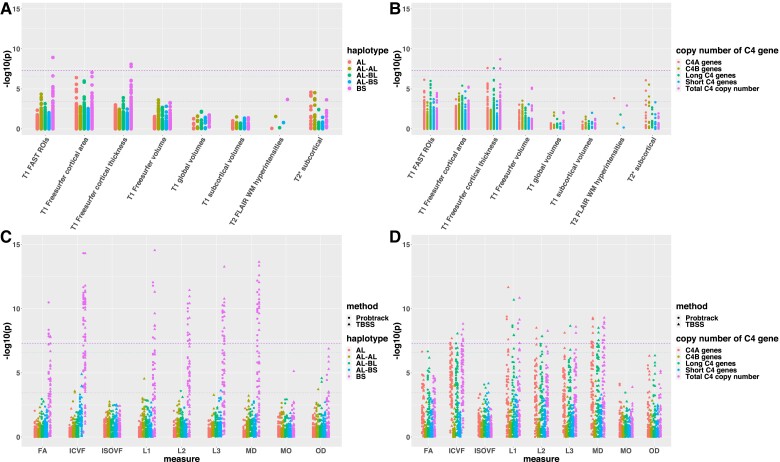
**The associations of common C4 haplotypes and C4 copy number with structural and diffusion IDPs**. The IDP groups are arranged on the *x*-axis as in Fig. 2. The strength of association (−log_10_(*P* value)) for individual IDP within each subgroup is plotted along the *y*-axis. The purple dash line indicates the genome-wide significant −log_10_(*P* value) threshold of 7.3. The blue dash line indicates the conservative −log_10_(*P* value) threshold of 6.6 accounting for the number of loci (137) as well as total IDPs (1422) tested. The grey dash line indicates a nominal significance −log_10_(*P* value) threshold of 3.44 correcting only for the number of the tested loci (137). Different structural haplotypes, C4 copy numbers are shown in different colours. Two statistical approaches (Probtrack and Tract-Based Spatial Statistics (TBSS)) for estimating dMRI measurements are distinguished by circle and triangle points. (**A**) The PheWAS plot of the association between distinct C4 haplotypes and structural IDPs. (**B**) The PheWAS plot of the association between C4A, C4B, C4L, C4S and total C4 copy numbers and structural IDPs. (**C**) The PheWAS plot of the association between distinct C4 haplotypes and diffusion IDPs. (**D**) The PheWAS plot of the association between C4A, C4B, C4L, C4S and total C4 copy numbers and diffusion IDPs.

The PheWAS plot for the associations of each common C4 haplotype to IDPs is presented in Fig. 6A and C. Five C4 haplotypes including C4-BS, C4-AL, C4-AL-AL, C4-AL-BL and C4-AL-BS were tested with IDPs. We identified a large number of associations between C4-BS and IDPs (Supplementary Table 10). The shared associations of C4-BS and the *HLA* alleles in AH8.1 across IDPs were due to the LD between them (Fig. 5). To be specific, the strongest association with structural IDPs was observed between C4-BS and grey matter volume in left thalamus (*P* = 1.18 × 10^−9^, beta = −0.068). For the associations between C4-BS and dMRI IDPs, we found significant positive associations with FA, ICVF and OD but negative associations with MD across multiple white matter tracts. No significant associations were found with ISOVF.

### The associations of *C4* gene copy number with IDPs

The two distinct isotypes of human *C4* gene, *C4A* and *C4B*, vary in copy number and structure, with the expression of each gene being proportional to gene copy number.^13^ The most significant associations across structural IDPs were observed between the total *C4* copy number and the mean TH of the left insula (*P* = 1.99 × 10^−9^) and long insula gyrus (*P* = 2.78 × 10^−8^), which is a small region and functionally regulates neuro-immune homeostasis.^47^ At the nominal significance threshold, we observed higher *C4A*, *C4L* and total *C4* copy number were associated with thinner regional cortical TH and decrease of grey matter in multiple regions, including frontal medial cortex and temporal fusiform cortex. In terms of the dMRI IDPs, we found higher *C4A*, *C4L* and total *C4* copy number were associated with lower FA, ICVF and OD and higher MD of multiple major white matter tracts, including posterior thalamic radiation, sagittal stratum and superior longitudinal fasciculus (Fig. 6B and D; Supplementary Table 17). No significant associations were found with ISOVF and MO. Considering *C4A* overexpression, but not *C4B*, increased synapse pruning,^48^ we also tested the associations of predicted *C4A* expression with IDPs. The predicted *C4A* expression level was calculated from the imputed haplotypes as previous work.^16^ Similar association patterns were observed with the associations to *C4A* copy number (Supplementary Fig. 9). These findings suggest *C4* expression level may not only affect grey matter but white matter microstructure within the brain.

### Limited associations were found between KIR and KIR/HLA pairs and IDPs

To identify the association between *KIRs* and IDPs, we examined the copy number of 17 *KIR* genes as well as the KIR haplotypes. No associations passed the genome-wide threshold. Considering the glycoprotein products encoded by the *KIR* genes are the receptors of the HLA class I ligands, we also tested the epistatic effects of KIR/HLA on IDPs (see ‘Materials and methods’ section). The most significant association was between KIR3DL1/HLA-B*27:02 and the volume of fifth ventricle under the dominant model (*P* = 4.21 × 10^−9^). The detailed results are in Supplementary Note and Supplementary Tables 18, 22–23.

### Multi-trait association analysis

The IDPs derived from the same MRI image or brain biomarker show phenotypic correlations (Supplementary Fig. 10). To increase the statistical power of detecting image-specific associations and address the phenotype redundancy problem, we performed multi-trait association analysis using TATES.^33^ We identified 90 associations surviving a Bonferroni correction for the number of immune variants and IDP groups (0.05/(137×19) = 1.9×10^−5^), and a further 67 associations between single genetic variants and 19 IDP groups when correcting for only the number of genetic variants (*P* < 3.6×10^−4^; Supplementary Table 24). This strategy can avoid multiple testing burden especially using the large number of IDPs and identify additional associations. For example, HLA-DRB1*01:01 was found to be associated with brain subcortical volumes, while HLA-C*07:02, HLA-B*07:02 and HLA-C*05:01 were significantly associated with T2 star metrics. As expected, we observed distinct genetic variants associated with different classes of IDPs. The *HLA* alleles in AH8.1 haplotype and *C4* copy number variation were found to be associated with multiple classes of IDPs, such as grey matter volumes, cortical TH, dMRI FA, ICVF and MD. In contrast, HLA-DRB1*01:01 and HLA-DQA1*01:01 were only associated with brain subcortical volumes. This suggested some of the genetic variants affect multiple groups of IDPs, whereas some are specific to single IDP group.

## Discussion

We conducted an association analysis of brain imaging phenotypes with the key immune genes, including *HLA*, *C4*, *KIR* as well as the interactions between *HLA* and *KIR*. The *HLA* locus has been associated with a number of neuropsychiatric and neurological diseases, such as schizophrenia, Parkinson’s disease, MS and Alzheimer’s disease. Our study complements genetic and neuroimaging studies of disorders of the brain in that it generates new insight into the relationship between immune related genes and brain MRI data which can help accelerate the discovery of new brain biomarkers.

Here, we identified a large number of associations between human immune-related genes and brain MRI-based phenotypes. We report 299 associations (*P* < 5 × 10^−8^) between immune related genes and IDPs, suggesting the immune-related genetic loci shape brain structure. These genetic associations were mainly identified with structural and dMRI phenotypes. Limited associations were observed with the fMRI data. The *KIR* locus was associated with a few phenotypes including white matter tracts in cerebrum (5 × 10^−8^ < *P* < 3.6 × 10^−4^). Only a small number of epistatic associations were identified between HLA/KIR interactions and IDPs at the nominal significance threshold. Larger samples are needed to gain sufficient power in future study.

Our results can help elucidate how the immune related variants impact specific brain structure, some of which have previously been implicated in disorders of the brain. Notably, HLA-B*08:01, HLA-DRB1*03:01 and HLA-DQB1*02 which are a part of the AH8.1 haplotype, relating to regional grey matter volumes, cortical area and TH in our study have been found to be associated with schizophrenia risk.^16^ These three alleles were also the most significant HLA allele that associated with major depression with modest effect size.^38^ Parkinson’s disease was reported to be associated with HLA-DRB1*04 and HLA-DQA1*03:01.^17,18,44^ Our findings could help unveil the mechanisms through which the HLA haplotypes contribute to neuropsychiatric and neurological diseases.

The *HLA* alleles within the AH8.1 haplotype were associated with most dMRI metrics except the ISOVF measures. In contrast, HLA-DRB1*04, HLA-DQB1*03:02, HLA-DQA1*03:01 and *HLA-DRB4* were only significantly associated with ISOVF in multiple white matter tracts (Fig. 2B; Supplementary Fig 6H–J). ISOVF is thought to measure the extra-cellular water diffusion.^49^ One possible interpretation is that the MHC class II molecules present the exogenous antigens to the CD4^+^ cells (T helper cells) compared to the MHC class I molecules present the intracellular peptides to the CD8^+^ cells (killer T cells).^50^ However, AH8.1 haplotype includes both class I and class II genes that exhibit strong LD. Thus, it is difficult to identify the causal variants.

The AH8.1 has been associated with many autoimmune disorders.^51^ Type 1 diabetes was reported to be associated with the component alleles in AH8.1, with HLA-DQB1*02:01 and HLA-DRB1*03:01 increased the risk of type 1 diabetes. These alleles shared associations with lupus, coeliac disease and sicca syndrome.^52^ The most significant associations of HLA-DQB1*02:01 and HLA-DRB1*03:01 were found with a decreased grey matter volume in the left thalamus. However, whether the autoimmune disorders share the association with brain morphology especially in some specific brain regions is largely unknown.

The higher *C4A* copy number has been found to be associated with higher schizophrenia risk, while the C4-BS allele is a protective factor of schizophrenia.^16^ A recent study further demonstrated the overexpression of *C4A* gene promotes the excessive synaptic loss and abnormal behaviour in mice.^48^ Interestingly, they found that the loss of *C4* does not affect the normal developmental synaptic pruning.^48^ At the nominal level of the significance threshold, C4-BS was associated with the increased cortical TH and grey matter volume across different brain regions especially the prefrontal cortex. The deficits of grey matter volume in prefrontal cortex were reported for some psychiatric disorders such as schizophrenia,^5^ bipolar disorder^53,54^ and major depression.^54^ Our findings may suggest C4-BS is a protective factor of the development of these diseases. On the other hand, we observed C4-BS was associated with higher FA, ICVF and OD but lower MD across multiple white matter tracts. Previous literature demonstrated the associations of ageing with microstructural connections within the white-matter quantified by using dMRI.^55^ Their results showed ageing was significantly associated with lower FA, ICVF and OD, with higher MD and ISOVF. Combined with our results, it suggests that C4-BS might be a protective factor of brain ageing. The strong LD between C4-BS and AH8.1 makes it difficult to find out the causal factor. However, the evidence that C4 does not play a role in normal development of cortical synapses^48^ suggested other mechanisms exist. One possible mechanism is through the MHC class I molecules which have been demonstrated to play a role in synaptic pruning during brain development.^41,56^ Previous findings implicated the expression and function of MHC I molecules in the regulation of synaptic plasticity, as well as their role in the aged brain.^42^ Future studies are needed to further understand each specific gene function, when and where they play a role. This requires looking at dynamic changes of brain spatial-temporal molecular characteristics using integrative genomics data.

We observed *C4A*, *C4L* and total *C4* copy numbers to be associated with many IDPs. For example, higher *C4A* copy number was associated with thinner cortical TH in some regions (Supplementary Table 17). Higher *C4A* copy number correlates with higher *C4A* expression.^16^ This may suggest that increased *C4A* expression plays a role in shaping the grey matter structure. In line with the findings of *C4* in schizophrenia, the associating thinner regional cortical TH might indicate increased expression of *C4A* as a disease mechanism. In addition to the associations with grey matter, the *C4A* copy number was also associated with the measures in dMRI across multiple white matter tracts. The alterations of the structure of white matter have been associated with many neurological diseases.^57–59^ But whether *C4* affects the risk of other diseases like Parkinson’s disease, amyotrophic lateral sclerosis has not been investigated yet using large-scale genetic data. Our results provide new insights into the potential role of *C4* on brain endophenotypes.

The KIR complex encoding the receptors of the MHC class I ligands is understudied in neurological diseases. These receptors including stimulatory and inhibitory normally work along with their corresponding MHC class I molecules. The first neurological disease found to be associated with KIR/HLA pairs was MS.^60^ Using the genetic epistatic model, we found only a small number of associations reached the nominal threshold. It is still not clear whether the interactions between *KIR* and *HLA* have influences on brain-related traits. Further exploration using clinical diseases as phenotypes is a direction of future study.

It is worth noting that the shared genetic associations between brain-MRI phenotypes and clinical diseases might be driven by the same brain regions. For example, we identified AH8.1 haplotype and predicted *C4A* gene expression level to be associated with brain imaging phenotypes, including grey matter volume in thalamus, cortical TH of insula and white matter structure in the superior longitudinal fasciculus. These genetic variants and brain regions were also reported to be associated with schizophrenia.^16,61–63^ Interestingly, insula is thought to play a role in brain-immune interaction.^47,64^ Loss of grey matter in insula was found to be associated with schizophrenia.^65^ HLA-A*03:01∼HLA-B*07:02 haplotype was found to be associated with Alzheimer’s disease. In our study, we identified the association between HLA-B*07:02 and T2* in hippocampus, which is affected at the early stage in Alzheimer's disease. Overall, our study contributes to identifying important brain regions associated with both immune genes and brain related diseases.

Our work still has several limitations. These association results were based on the imputed genetic variants. The *HLA* alleles can be imputed accurately given that many associating *HLA* alleles have been replicated well. However, the accuracy of the *KIR* imputation can be sensitive to the overlapping SNPs between the genotyping data and the reference panel. We controlled for several known or putative imaging confounders in the association analysis, following some recent UKB analyses, though other unknown of unmeasured confounders may exist. In addition, the long-range LD patterns in the HLA region make it difficult to fine-map the causal variants. A comprehensive genetic map as well as functional annotations is needed to improve the understanding of the disease mechanisms caused by this complex locus. We did not replicate these associations due to the lack of brain imaging data from other independent cohorts. Lastly, only European descents were included in our study because of the limited representation of participants from different ancestry backgrounds in UK Biobank imaging study. Despite these limitations, our study generates new insights into the immunogenetics of human brain structure.

## Supplementary Material

fcac078_Supplementary_Data

## Abbreviations

AH8.1 =: ancestral haplotype 8.1
C4 =: complement component 4
HLA =: human leukocyte antigen
IDP =: image-derived phenotype
KIR =: killer cell immunoglobulin-like receptor
MHC =: major histocompatibility complex
PheWAS =: phenome-wide association study
SNP =: single nucleotide polymorphism
TATES =: trait-based association test that uses extended Simes procedure
