# Supplementary material for: The role of critical immune genes in brain disorders: insights from neuroimaging immunogenetics
Source: Brain Commun. 2022 Mar 31;4(2):fcac078. doi: 10.1093/braincomms/fcac078 (PMC9014537; doi:10.1093/braincomms/fcac078)
Supplement: fcac078_Supplementary_Data [file fcac078_supplementary_data.zip › Supplementary_Materials.docx]

**Supplementary Figures**

**Supplementary Figure 1.** The distribution of four IDPs exhibiting extreme skewness. Extreme outliers (greater or less than 15 times median absolute deviation from the median) were removed before plotting.


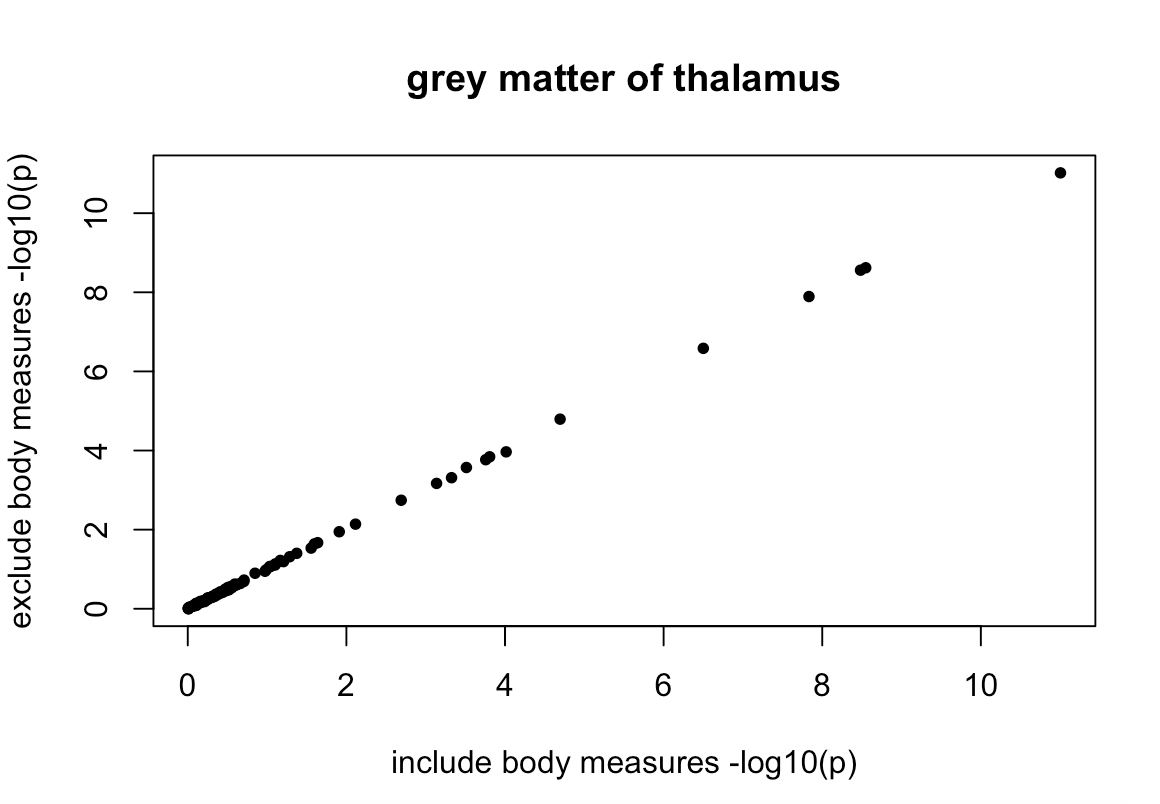


**Supplementary Figure 2.** The relationship between -log10(P) derived from two strategies. X-axis indicates -log10(P) derived with body measures included. Y-axis indicates -log10(P) derived with body measures excluded.


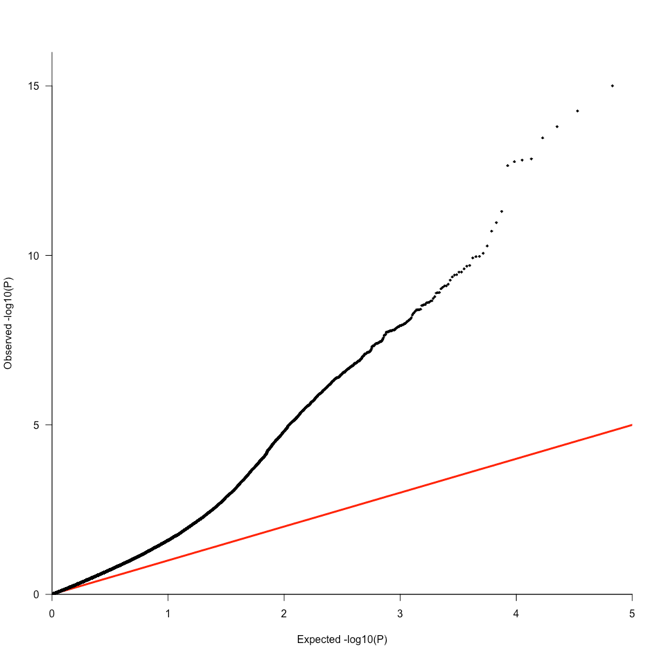
**
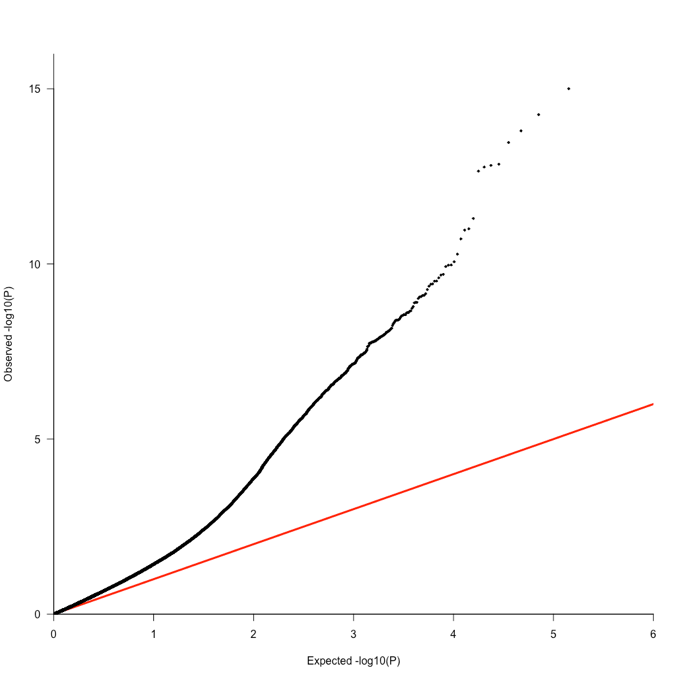
**

b

a

d

c

**
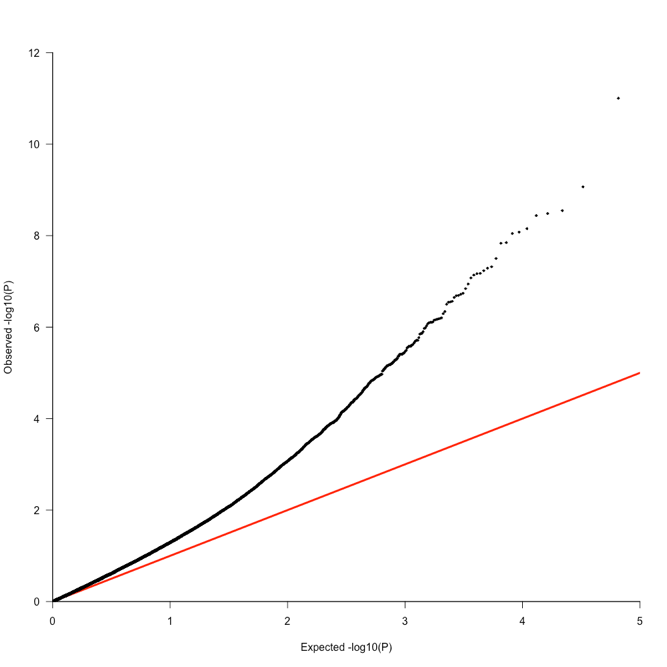
**
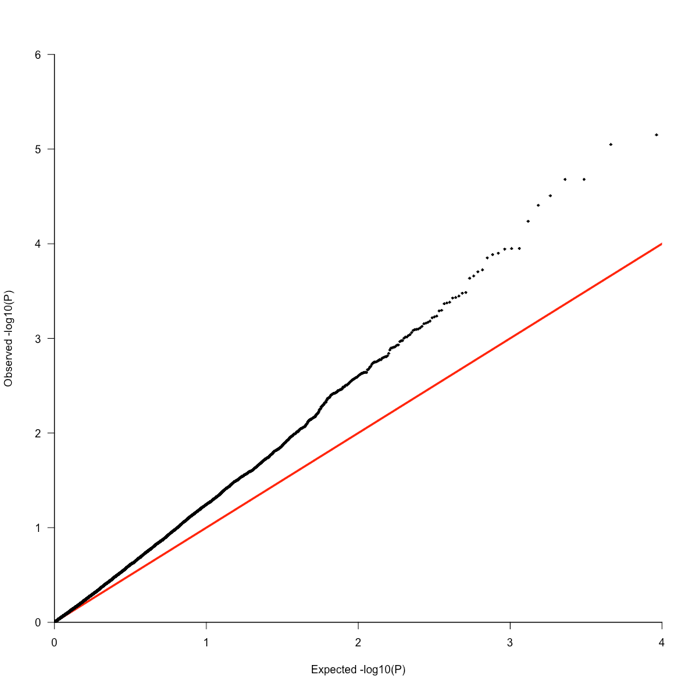


**Supplementary Figure 3.** (a) QQ-plot for the associations of HLA and all IDPs. (b) QQ-plot for the associations of HLA and structural IDPs. (c) QQ-plot for the associations of HLA and dMRI IDPs. (d) QQ-plot for the associations of HLA and fMRI (tfMRI and rfMRI nodes).


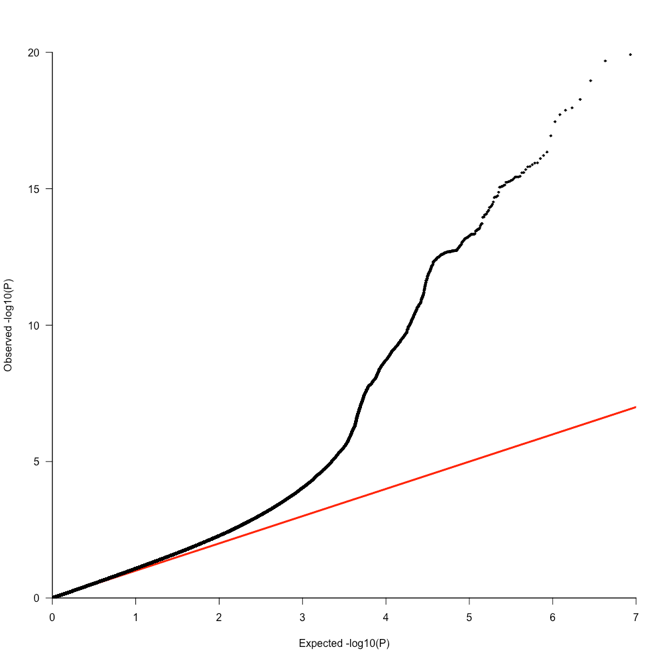

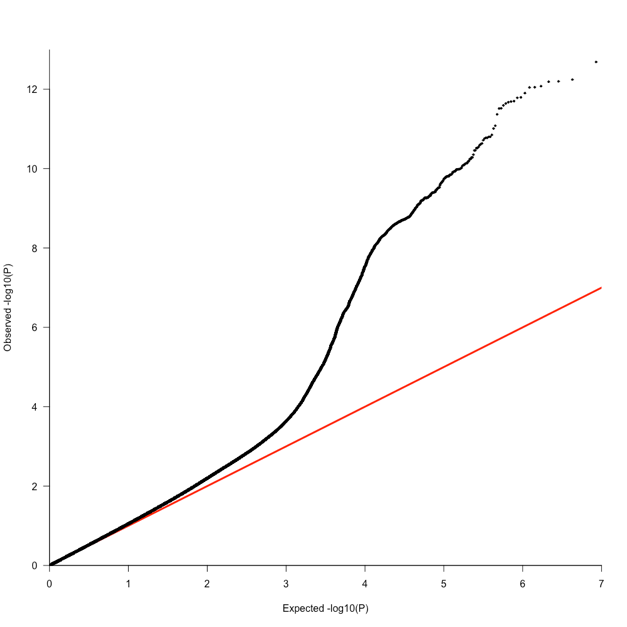


b

a

d

c


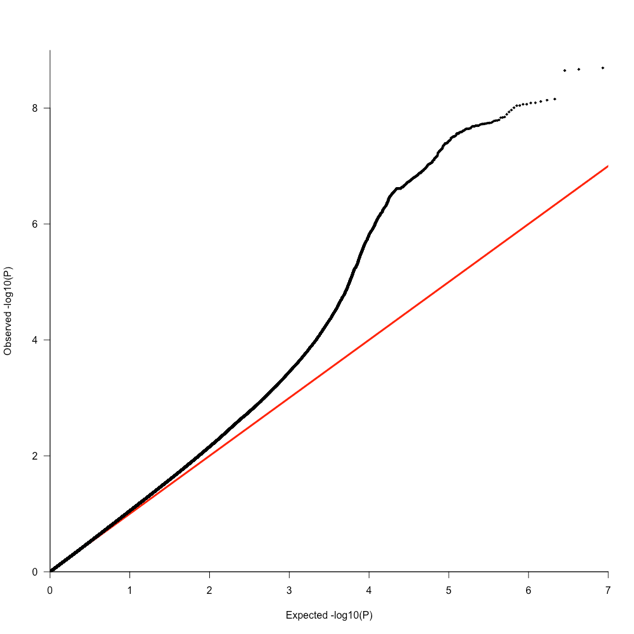

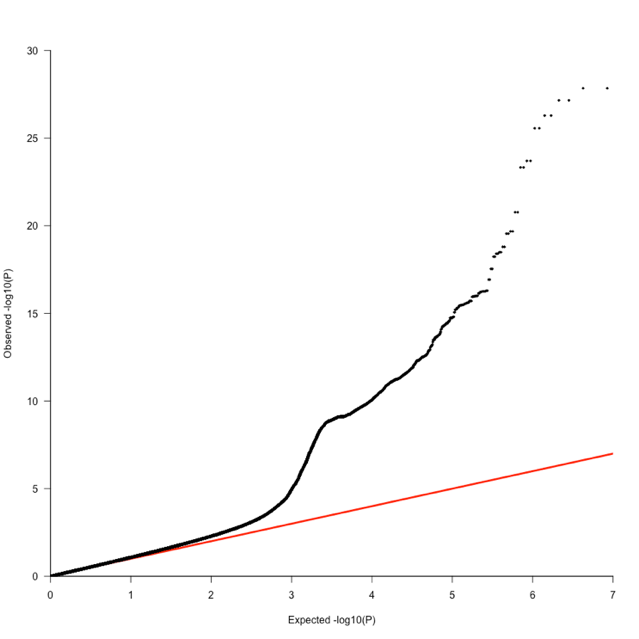


e

e


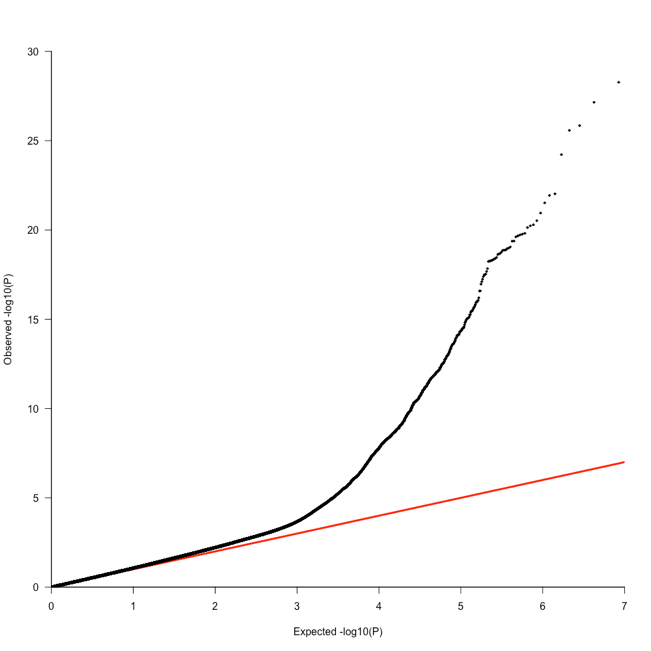


**Supplementary Figure 4**. QQ-plots of GWAS test statistics of five selected IDPs. X-axis is the expected -log10(P). Y-axis is the observed -log10(P). (a) QQ-plot of GWAS test statistics of volume of grey matter in Thalamus (left). (b) QQ-plot of GWAS test statistics of mean thickness of insula (left hemisphere). (c) QQ-plot of GWAS test statistics of mean thickness of posteriorcingulate (right hemisphere). (d) QQ-plot of GWAS test statistics of mean MD in superior corona radiata on FA skeleton (right). (e) QQ-plot of GWAS test statistics of Weighted-mean ISOVF in tract superior longitudinal fasciculus (right).

**Supplementary Fig 5. PheWAS plot of the association between the selected HLA alleles and structural IDPs.** **a-k**, PheWAS plot of the association between HLA-A*01:01, HLA-DQA1*05:01, HLA-DRB1*03:01, HLA-DQB1*02:01, HLA-B*07:02, HLA-C*05:01, HLA-C*07:02, HLA-DRB4*01:03, HLA-DRB1*04:04, HLA-DQA1*03:01 and HLA-DRB3*01:01 and structural IDPs. Different subgroups of IDPs are given along the x-axis. The strength of association (-log_10_(P value)) for individual IDP within each subgroup is given on the y-axis. The purple dash line indicates the genome-wide significant -log_10_(P value) threshold of 7.3. The blue dash line indicates the conservative -log_10_(P value) threshold of 6.6 accounts for the number of loci (137) as well as total IDPs (1,422) tested. The grey dash line indicates a nominal significance -log_10_(P value) threshold of 3.44 correcting only for the number of the tested loci (137).

**Supplementary Fig 6. PheWAS plot of the association between selected HLA alleles and diffusion MRI IDPs.** **a-k**, PheWAS plot of the association between HLA-A*01:01, HLA-DQA1*05:01, HLA-DRB1*03:01, HLA-DQB1*02:01, HLA-B*07:02, HLA-C*05:01, HLA-C*07:02, HLA-DRB4*01:03, HLA-DRB1*04:04, HLA-DQA1*03:01 and HLA-DRB3*01:01 and diffusion IDPs. Different subgroups of IDPs are given along the x-axis. The strength of association (-log_10_(P value)) for individual IDP within each subgroup is given on the y-axis. Probtrack and TBSS represent two statistical approaches for DTI measures analysis. The purple dash line indicates the genome-wide significant -log_10_(P value) threshold of 7.3. The blue dash line indicates the conservative -log_10_(P value) threshold of 6.6 accounts for the number of loci (137) as well as total IDPs (1,422) tested. The grey dash line indicates a nominal significance -log_10_(P value) threshold of 3.44 correcting only for the number of the tested loci (137).


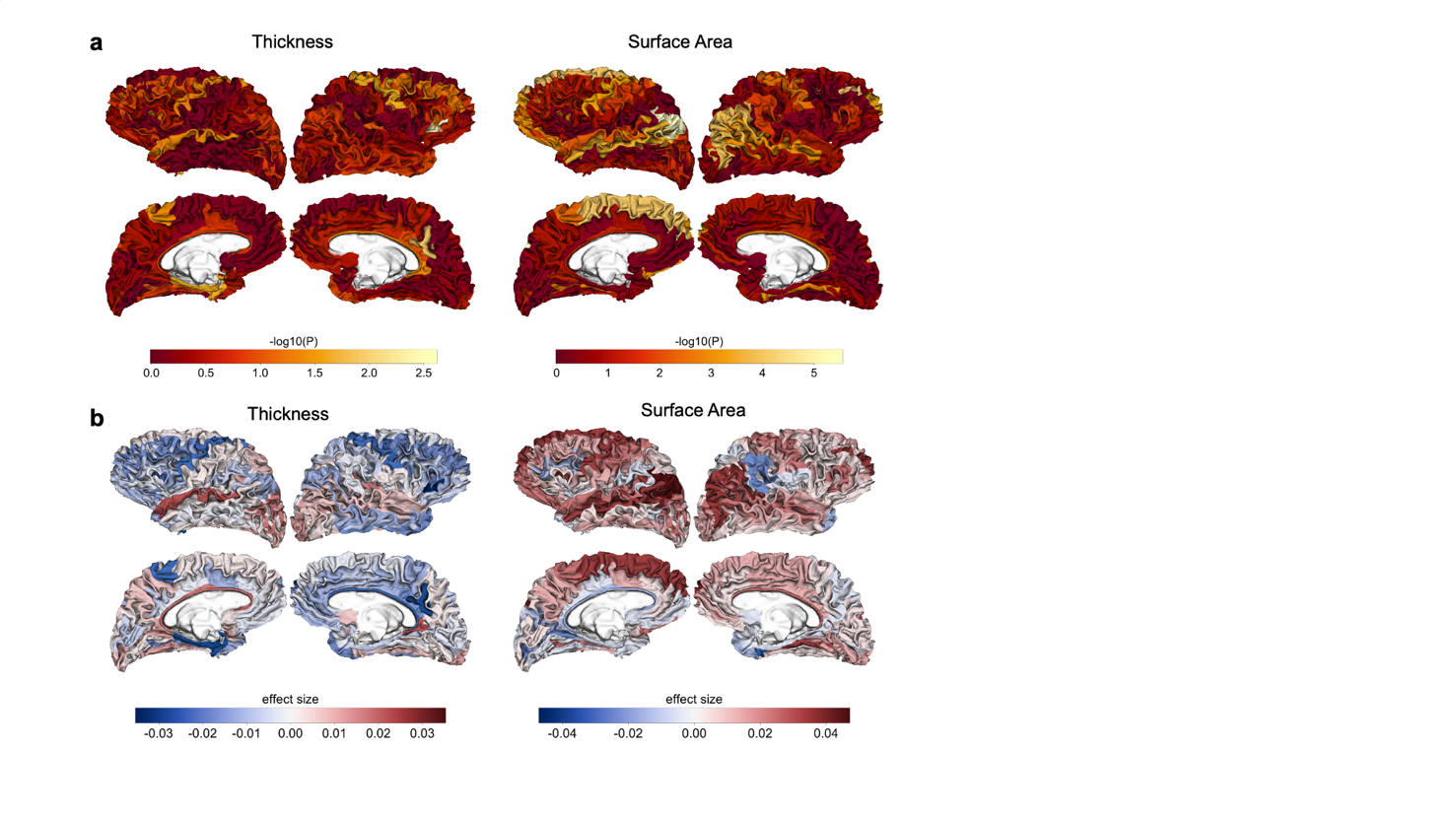


**Supplementary Fig 7. The associations between HLA-B*07:02 and cortical surface area (SA) and thickness (TH) within whole brain. a**, Spatial maps of the association between HLA-B*07:02 and SA and TH within whole brain. -log_10_(P values) are shown on freesurfer a2009s atlas with 74 regions included. High -log_10_(P values) are shown in yellow, while low -log_10_(P values) are shown in red. **b**, Spatial maps of the effect size of HLA-B*07:02 on TH and SA within whole brain. Positive effects are shown in red, while negative effects are shown in blue.


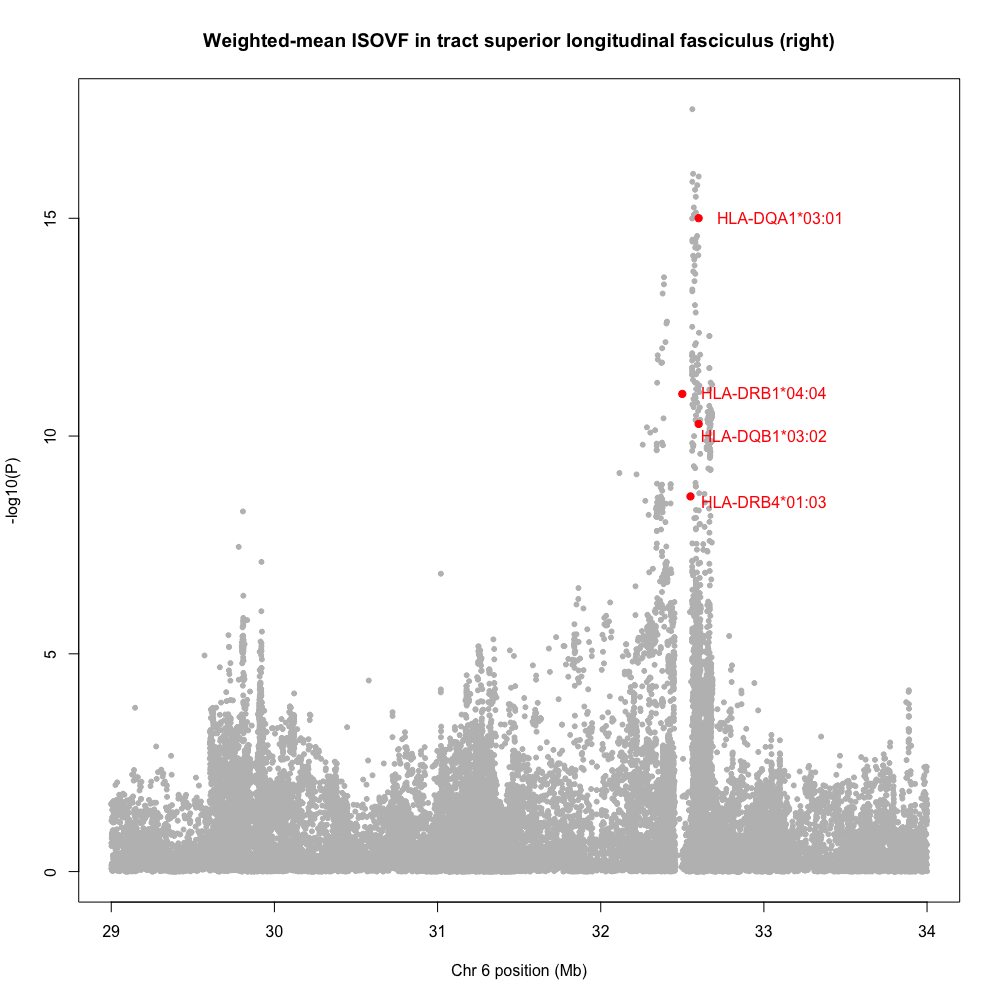


a


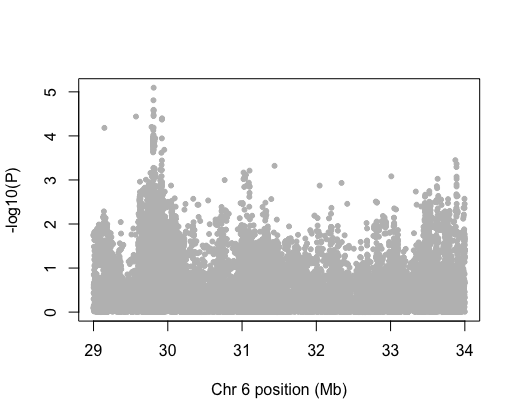


Controlling for DQ, DR genes


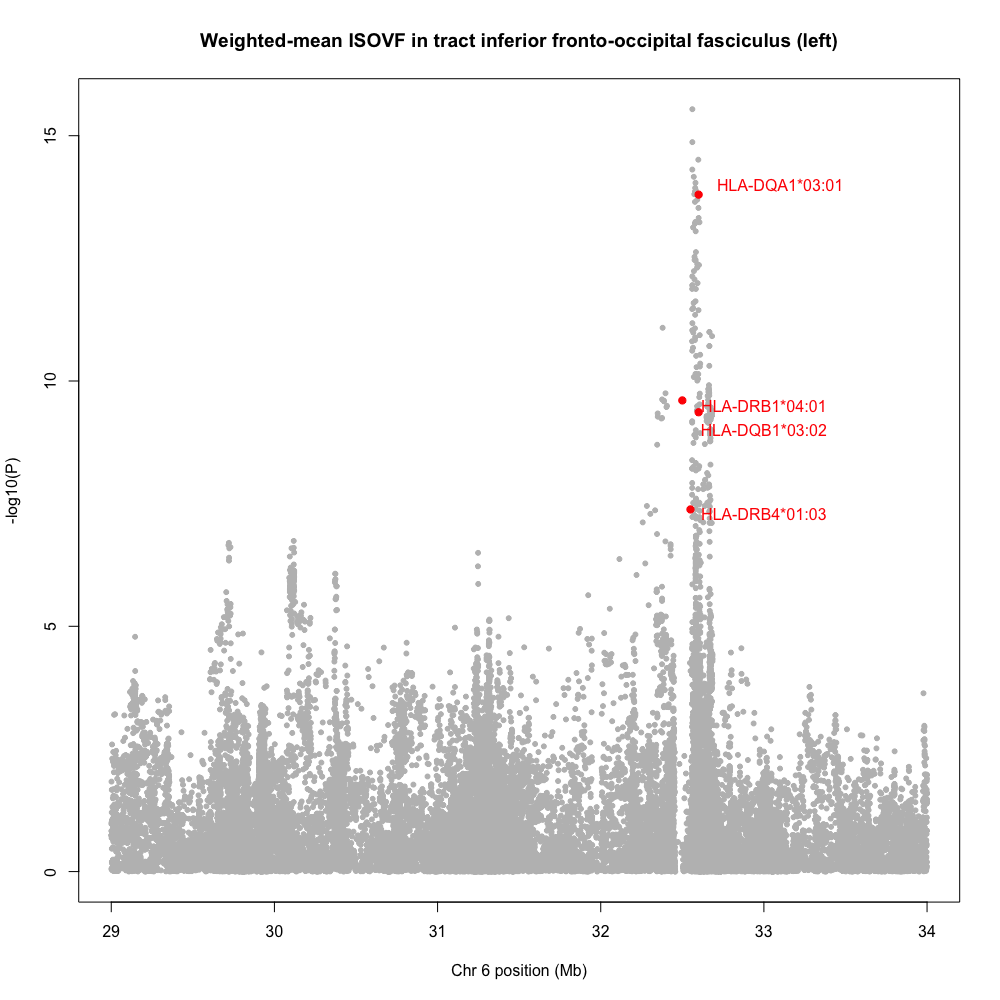


b


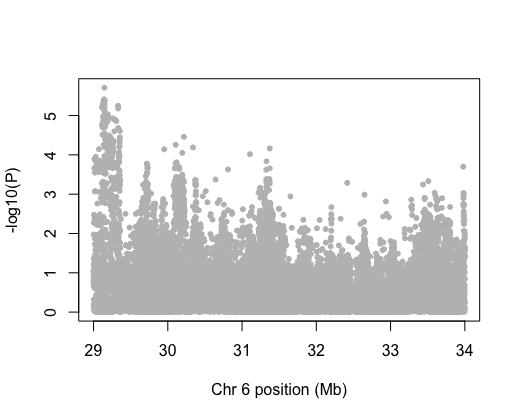


Controlling for DQ, DR genes


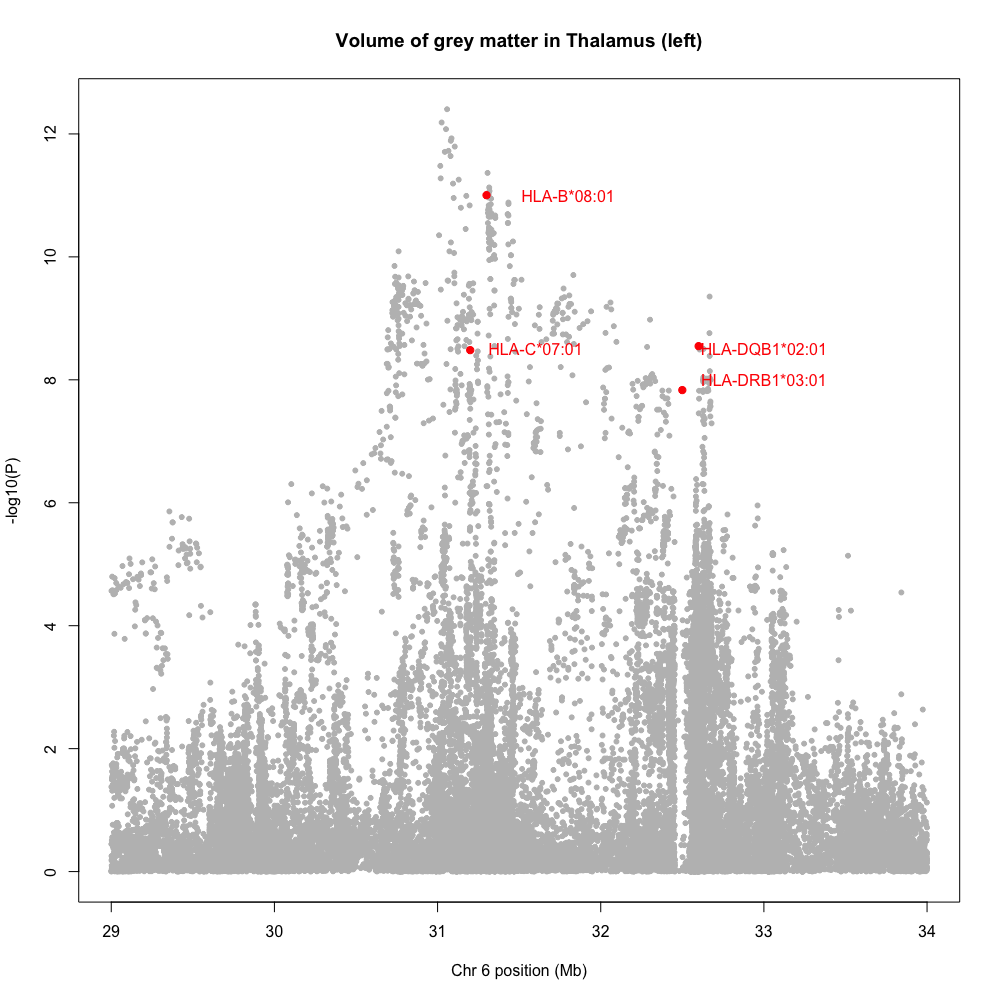


c


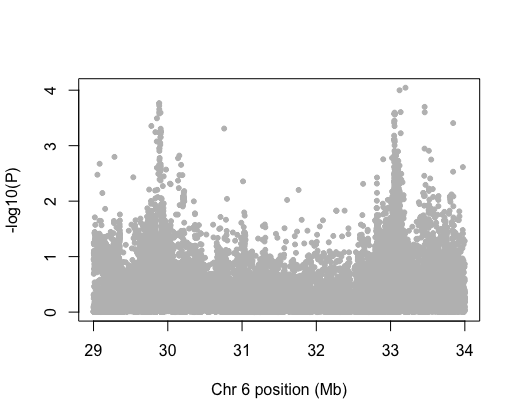


Controlling for B, C, DQ, DR genes


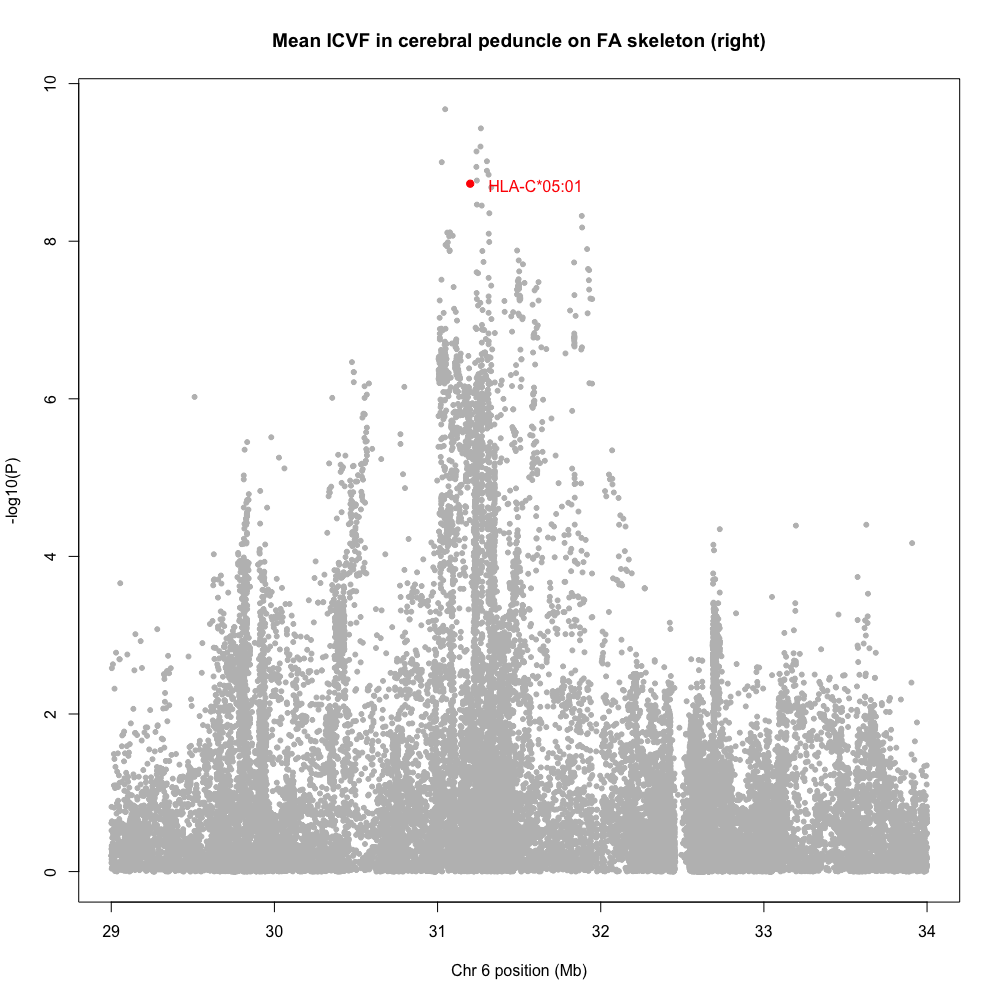


d


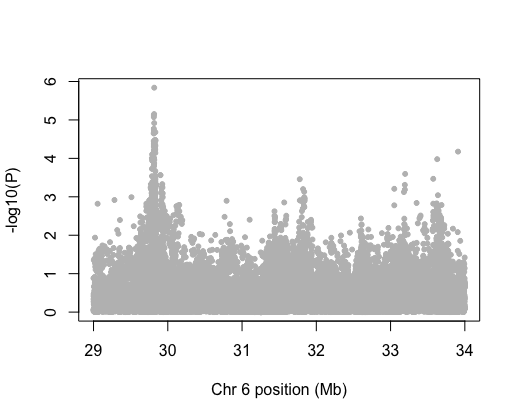


Controlling for C


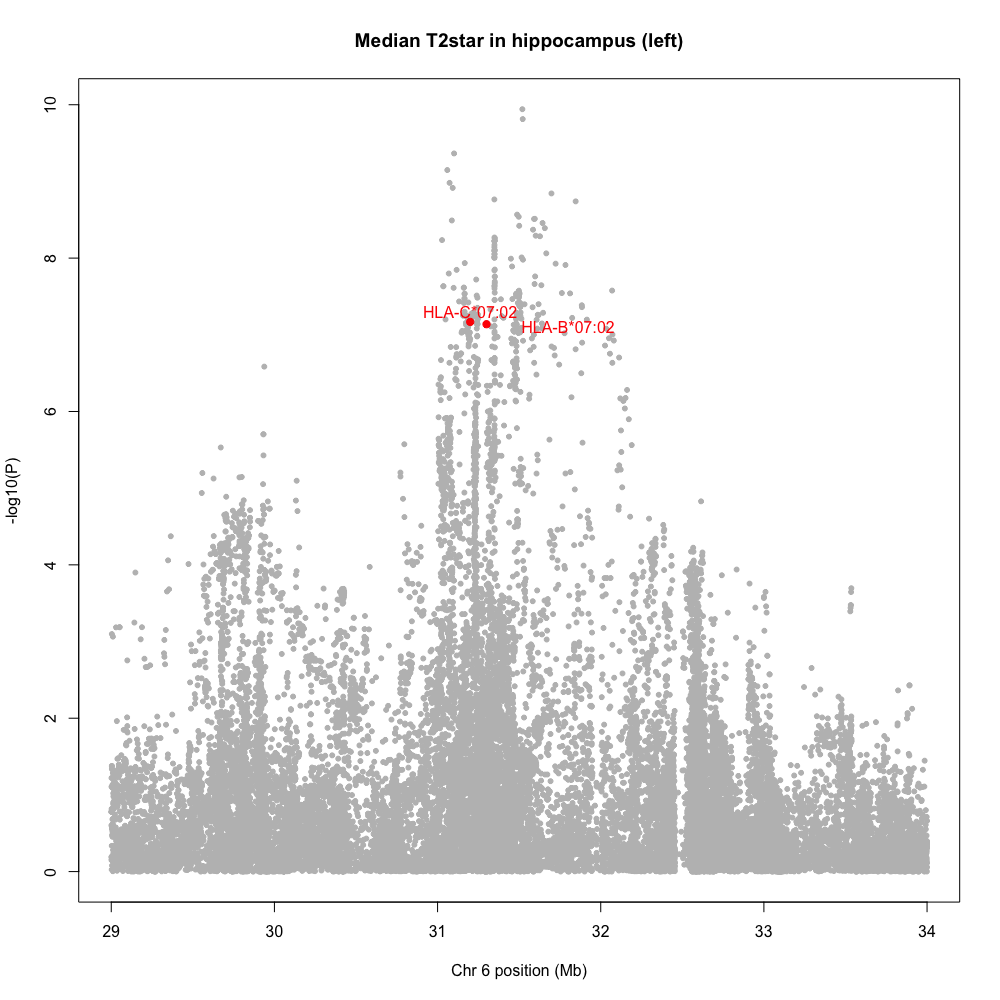


e


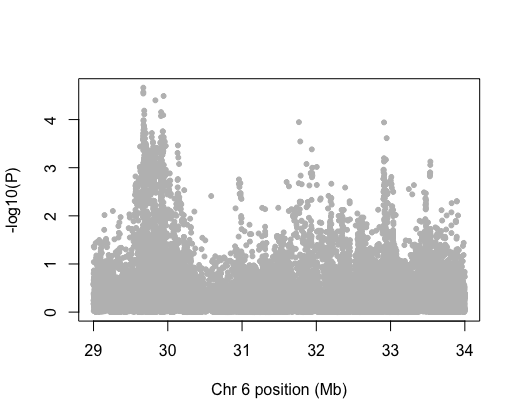


Controlling for C, B

**Supplementary Figure 8. Regional Manhattan plots of the five selected IDPs.** The top one is the regional association results. The bottom one is the regional association results after controlling for all the HLA alleles of the associating genes.

**Supplementary Fig 9. The strength of associations of predicted C4A expression with structural and diffusion IDPs.** The x-axis and y-axis are the same as in Fig 6. **a**, The PheWAS plot of the association between predicted C4A expression and structural IDPs. **b**, The PheWAS plot of the association between predicted C4A expression and diffusion IDPs. Two statistical approaches (Probtrack and TBSS) for estimating dMRI measurements are distinguished by circle and triangle points.

T1_FAST_ROIs T1_brain_volumes


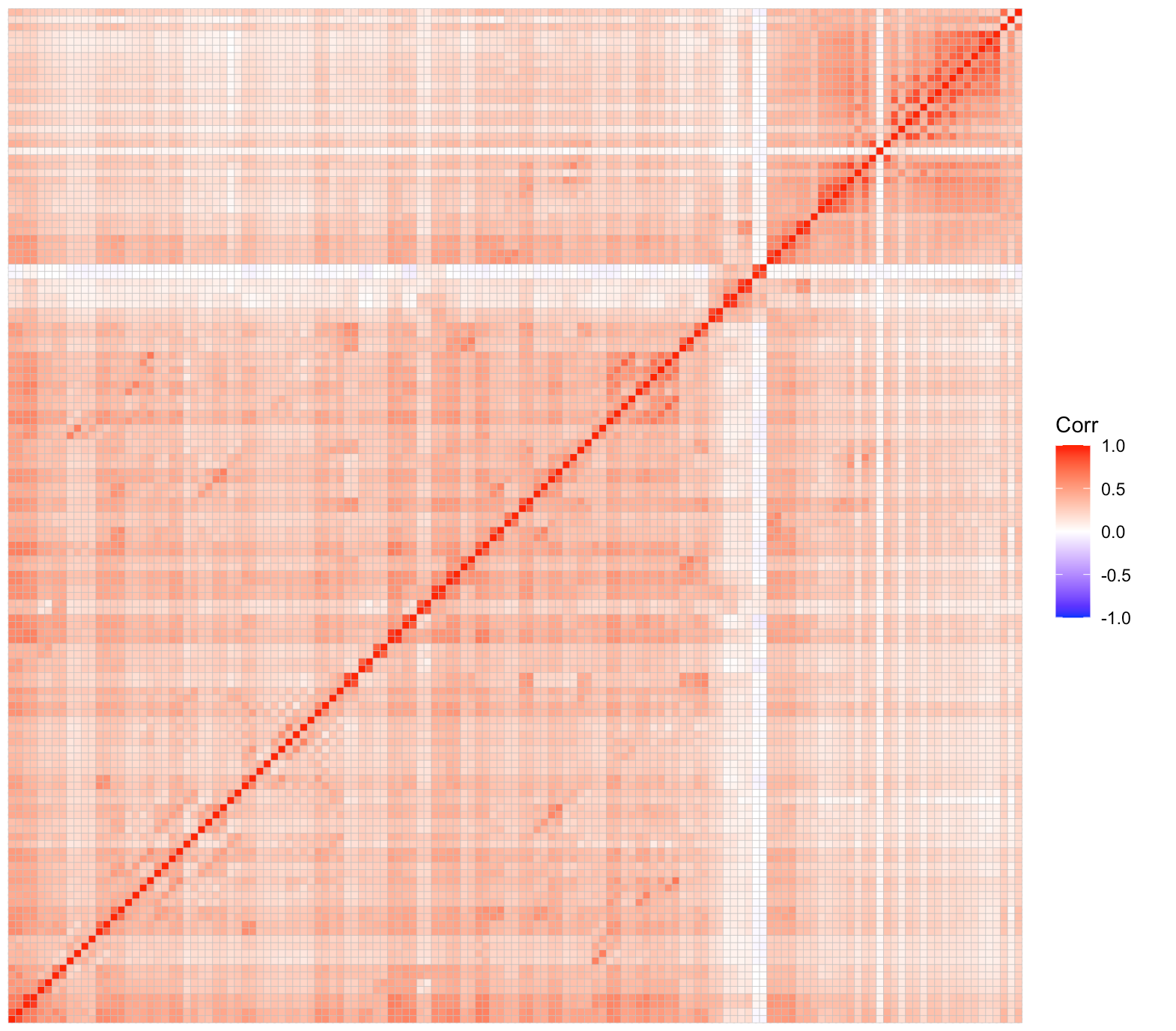

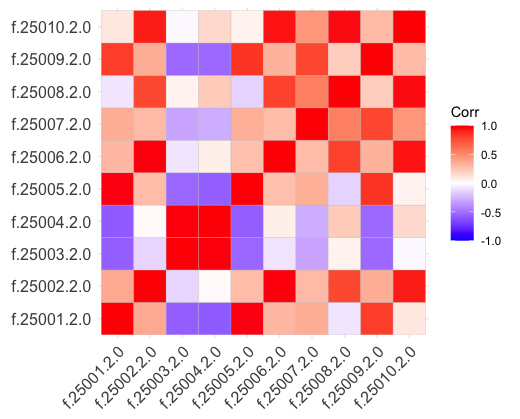


T1_subcortical_volumes FreeSurfer


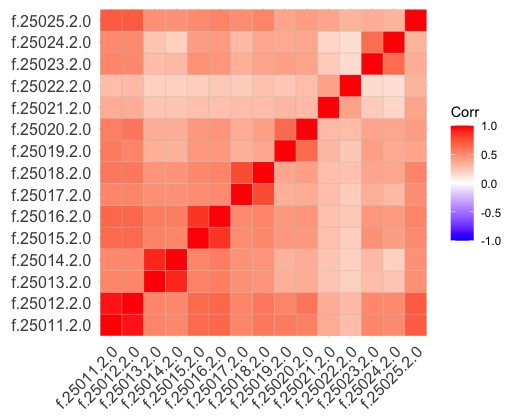

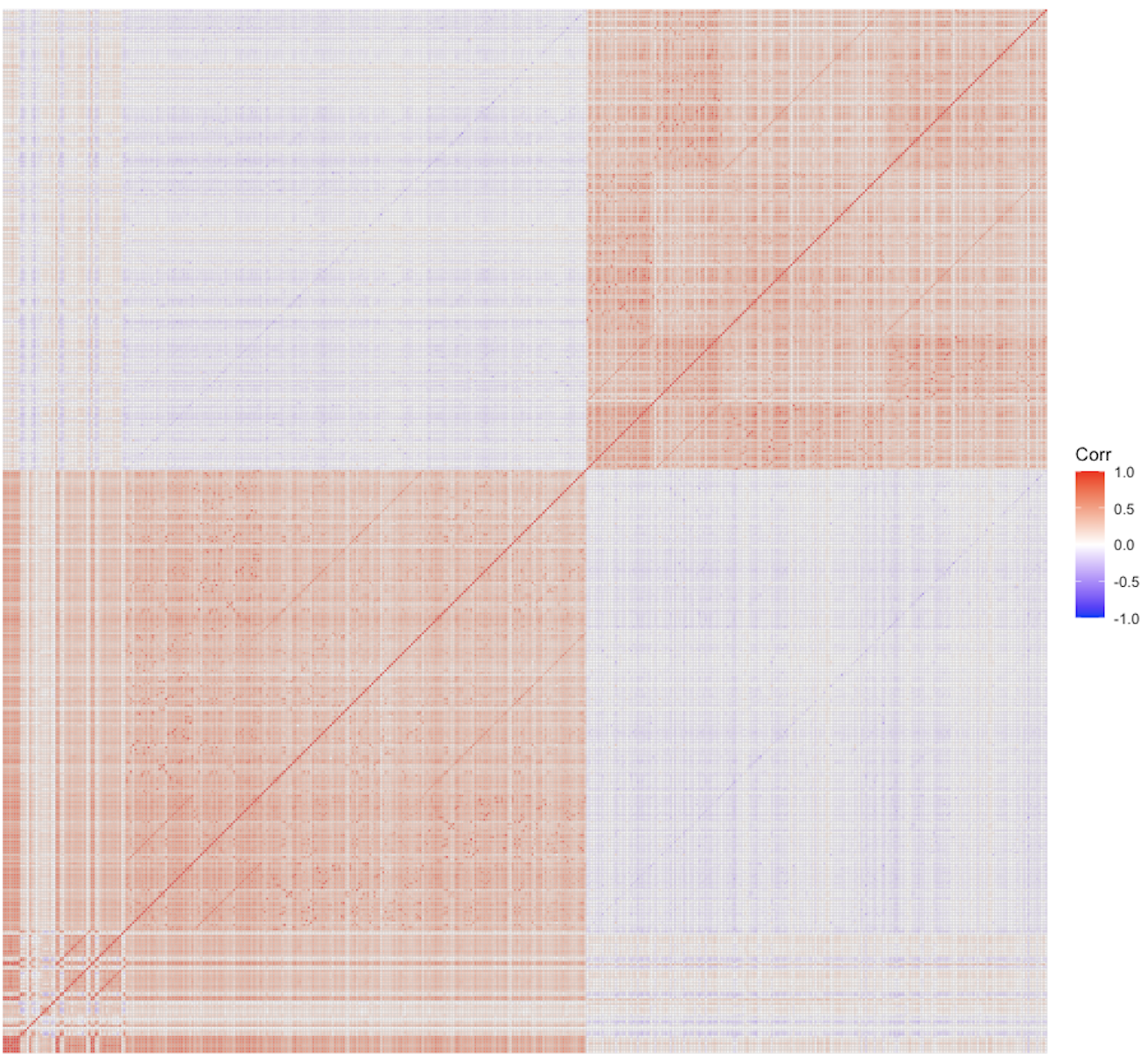


T2 Star tfMRI


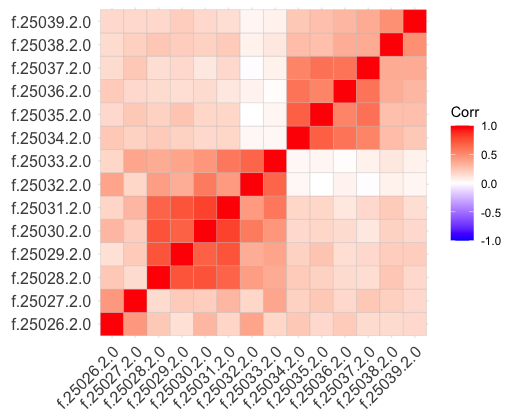

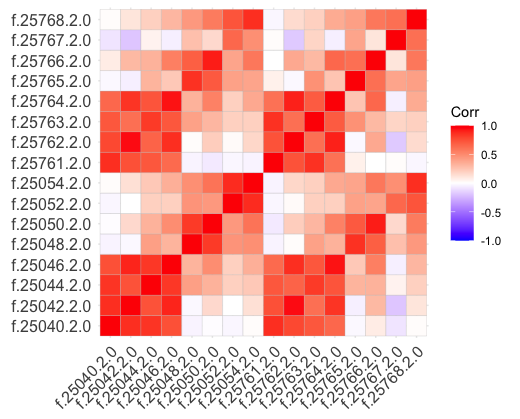


dMRI resting fMRI - parcellation25 amplitudes


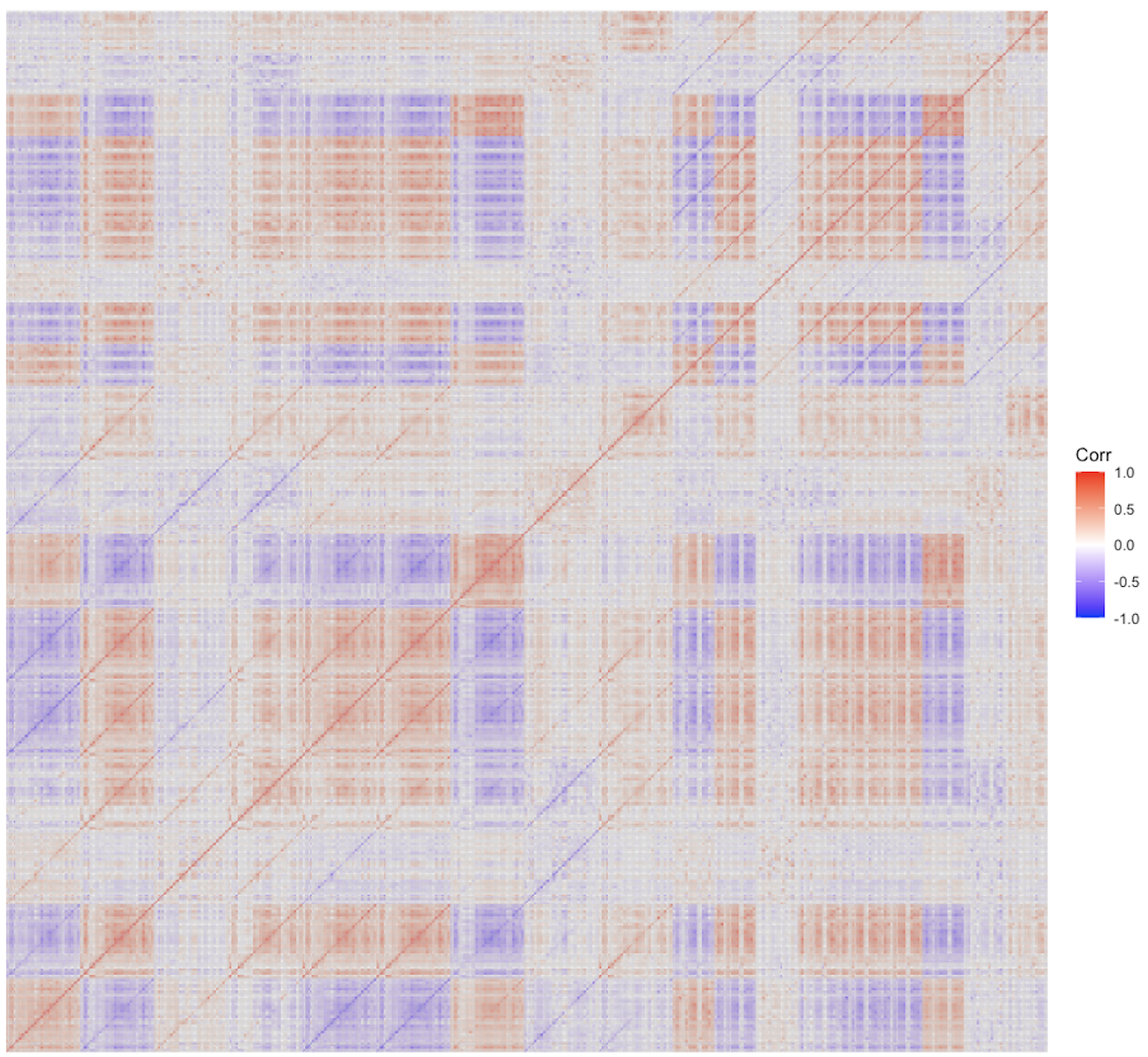

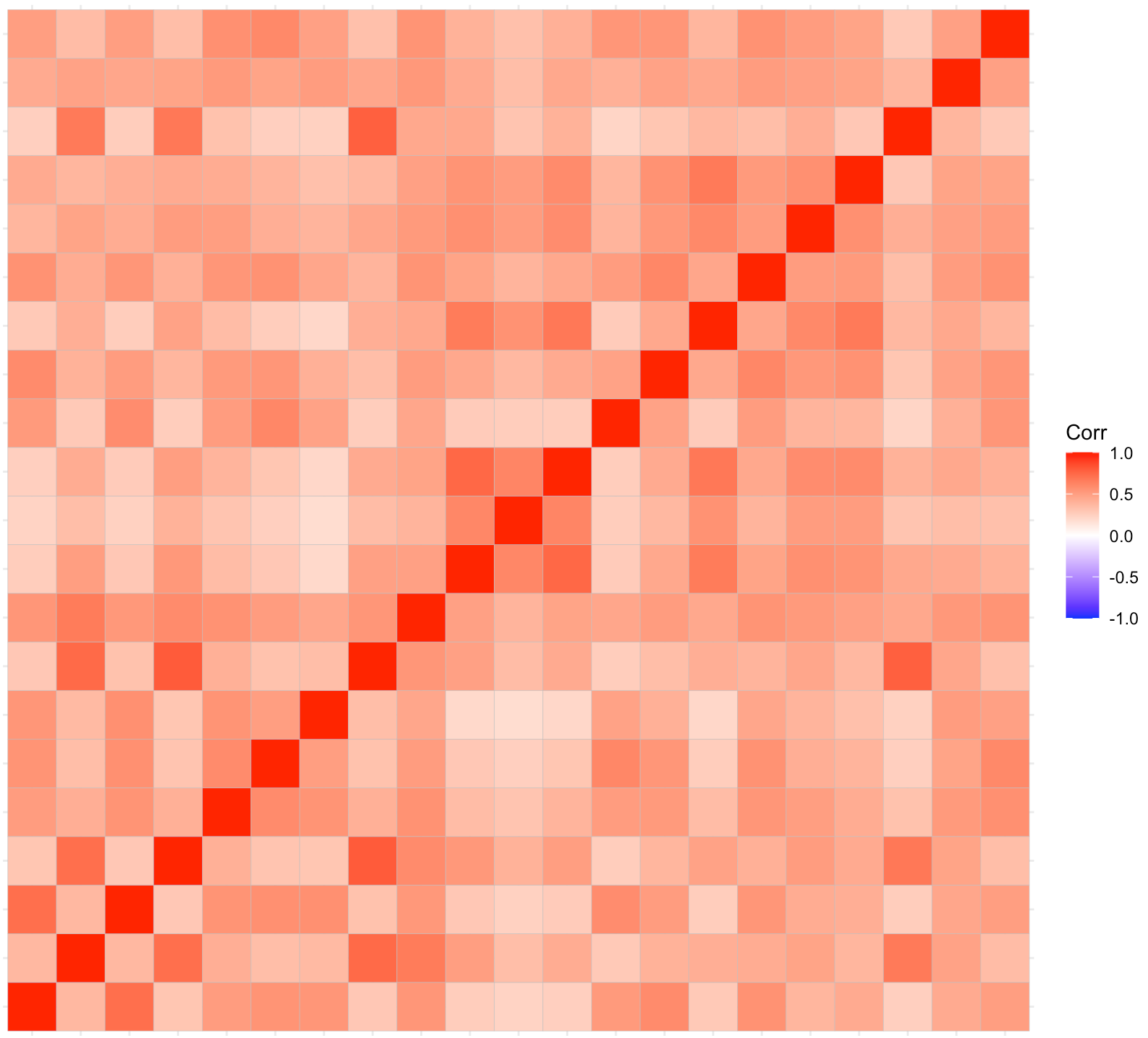


resting fMRI - parcellation25 amplitudes


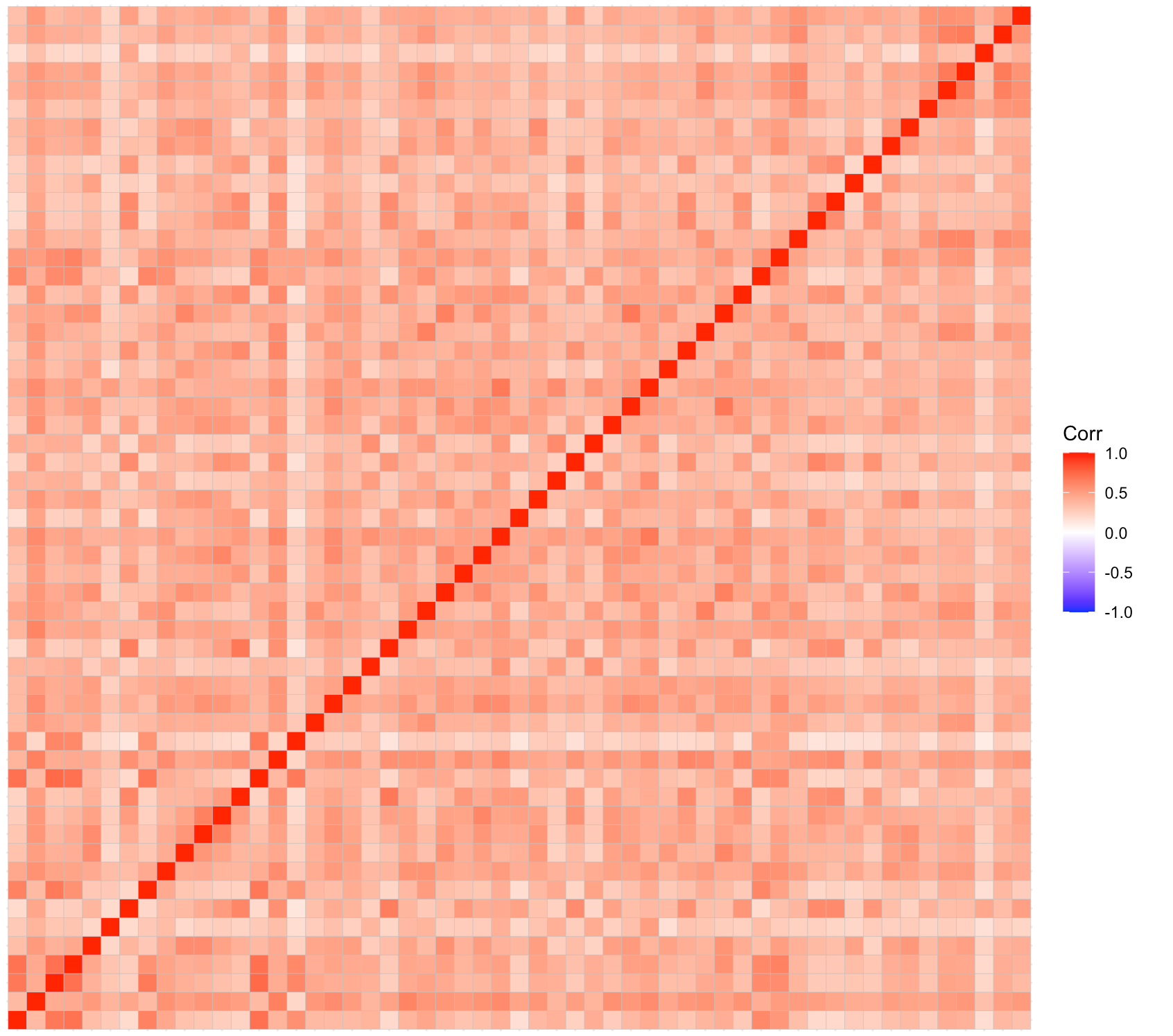


**Supplementary Fig 10**. The phenotypic correlation of different IDP groups.

**Supplementary Figure 11.** Pairwise LD between KIR genes calculated based on the KIR imputation results. The number in each cell represents R^2^.

**Supplementary Note**

1. **Reference panel for C4 imputation**

There are two consent groups in the released reference panel. We used the data for general research use, containing 1180 samples of mixed ancestry. We then removed 40 individuals that reflect duplicate samples or shared ancestry at this locus as suggested in their documentation. Therefore, 1140 samples were used for C4 imputation.

1. **The GWAS of 5 selected IDPs**

The genotype data were imputed by the UK Biobank team using the Haplotype Reference Consortium (HRC)^1^ and UK10K^2^ reference panels. We then followed the genotype quality control pipeline in Revez et.al, 2020^3^. We used 8,539,875 SNPs in total for GWAS. The same confounding factors were included as the HLA association study did. The GWAS analysis was carried out using BGENIE v1.2^4^.

1. **Supplementary results**

**Potential collider bias is limited.** The IDPs can be correlated with body measures^5^. Adjusting body measures could introduce collider bias. To assess the potential collider bias in our analysis, we selected grey matter of thalamus (left) which was found to be associated with HLA alleles as an example phenotype. We performed association tests without the body measures (height, weight and BMI) as covariates. The correlation of -log10(p-values) while including and excluding body measures was 0.999 (P=2e-127), indicating potential collider bias is limited (**Supplementary Figure 2**).

**KIR genes mainly related to the changes of white matter tracts.** To illustrate the relationship between KIR genes and IDPs, we conducted the association analyses using 19 KIR loci (including general and fine-scale haplotypes). We first calculated pairwise LD between the KIR genes (**Supplementary Fig. 11**). No associations passed the genome-wide significant threshold. At the threshold of 3.6×10^-4^, we observed 29 associations (**Supplementary Table 18**). Most of these associations pointed to dMRI IDPs. For example, the associations of KIR2DS4WT with dMRI measures were identified in some specific white matter tracts such as cerebral peduncle, posterior limb of internal capsule and retrolenticular part of internal capsule. We also found KIR2DS4WT was associated with white matter hyperintensities volume which was related to increased risk of ageing-related diseases^6^.

**Epistasis between KIR and HLA ligands across IDPs.** Killer immunoglobulin-like receptors (KIRs) bind to their MHC class I ligands to determine NK cell inhibition and activation. The associations between KIR/HLA ligand and disease outcome have been widely confirmed in the fields of virus and cancer but still remain largely unknown in neurological diseases. To investigate whether the epistasis between KIR genes and HLA genes contributes to brain-related traits, we tested different combinations of KIR and HLA genotypes under the KIR dominant and recessive model, respectively. 112 KIR-HLA pairs (**Supplementary Table 7**) were tested. No associations reached the threshold of 5×10^-8^ except the association of KIR3DL1/HLA-B*27:02 and the volume of 5^th^ ventricle under the dominant model (P = 4.21×10^-9^). We applied 0.05/112=4.5×10^-4^ to further highlight 55 associations under recessive model and 44 associations under dominant model, respectively (**Supplementary Tables 22-23**). It can be challenging to make interpretation with either KIR associations or epistatic associations not only due to the complexity of these immune genes but brain itself. Previous studies suggested the co-evolution of NK cell receptors and MHC-C molecules contributes to modern human brain development^7^. Further investigations at molecular level are needed to understand how the receptor-ligand system affects brain development.

**Supplementary References**

1. McCarthy, S. *et al.* A reference panel of 64,976 haplotypes for genotype imputation. *Nat. Genet.* **48**, (2016).

2. Walter, K. *et al.* The UK10K project identifies rare variants in health and disease. *Nature* **526**, (2015).

3. Revez, J. A. *et al.* Genome-wide association study identifies 143 loci associated with 25 hydroxyvitamin D concentration. *Nat. Commun.* **11**, (2020).

4. Bycroft, C. *et al.* The UK Biobank resource with deep phenotyping and genomic data. *Nature* **562**, (2018).

5. Couvy-Duchesne, B. *et al.* A unified framework for association and prediction from vertex-wise grey-matter structure. *Hum. Brain Mapp.* **41**, (2020).

6. Habes, M. *et al.* White matter hyperintensities and imaging patterns of brain ageing in the general population. *Brain* **139**, (2016).

7. Parham, P. & Moffett, A. Variable NK cell receptors and their MHC class i ligands in immunity, reproduction and human evolution. *Nat. Rev. Immunol.* **13**, 133–144 (2013).
